# Sequencing BCMA-Directed Therapies in Relapsed/Refractory Multiple Myeloma: A Real-World Comparison of CAR T-Cell Therapy and Bispecific Antibodies

**DOI:** 10.3390/cancers18193147

**Published:** 2026-09-29

**Authors:** Osama M. Younis, Sencer Goklemez, Carmel Awadallah, Shebli Atrash, Barry Paul, Hira Shaikh, Christopher Strouse, Andrew Vegel, Emily Struble, Anita Mazloom, Omar Alkharabsheh, Prerna Mewawalla, Mansi R. Shah, Abhiraj Saxena, Zahra Mahmoudjafari, Jordan Snyder, Muhammad Umair Mushtaq, Jeries Kort, Alma Habib, Joseph McGuirk, Al-Ola Abdallah, Abdullah Mohammad Khan

**Affiliations:** 1 School of Medicine, University of Jordan, Amman 11942, Jordan; 2 Division of Hematology and Oncology, University of Cincinnati, Cincinnati, OH 45267, USA; 3 St. John’s Episcopal Hospital, New York, NY 11691, USA; 4 Levine Cancer Center, Atrium Health, Wake Forest University School of Medicine, Charlotte, NC 28204, USA; 5 Division of Hematology, Oncology, and Blood & Marrow Transplantation, University of Iowa, Iowa City, IA 52242, USA; 6 Division of Hematology and Oncology, Mitchell Cancer Institute, University of South Alabama, Mobile, AL 36688, USA; 7 Division of Hematology and Cellular Therapy, Allegheny Health Network Cancer Institute, Pittsburgh, PA 15212, USA; 8 Division of Blood Disorders, Rutgers Cancer Institute, New Brunswick, NJ 08901, USA; 9 Division of Hematologic Malignancies & Cellular Therapeutics, University of Kansas Medical Center, Westwood, KS 66160, USA; 10 Division of Hematology, The Ohio State University, Columbus, OH 43210, USA

**Keywords:** relapsed/refractory multiple myeloma, CAR-T, bispecific antibodies, sequencing therapies

## Abstract

Background: Several B-cell maturation antigen (BCMA)-directed therapy (BDT) modalities have been approved by the US Food and Drug Administration (FDA) for relapsed-refractory multiple myeloma (RRMM). Ideal sequencing of these therapies remains unknown. Methods: We report one of the largest multicenter retrospective patient cohorts in this real-world analysis of patients receiving a BDT in both BDT-naïve and exposed settings at major academic centers. Results: In the BDT-naïve setting, which included 276 patients, 169 (61%) received chimeric antigen receptor T-cell therapy (CAR-T), whereas 107 (39%) received bispecific antibodies (BsAbs). Median progression-free survival (PFS) in the BDT-naïve setting was significantly longer with CAR-T than with BsAbs (16.1 vs. 13.5 months, *p* = 0.007) with a higher overall response rate (ORR) (87 vs. 68%). Multivariable Cox regression analysis revealed that receiving CAR-T (hazard ratio [HR] = 0.41), cytokine release syndrome (CRS) of any grade (HR = 0.50), achieving a best response of ≥very good partial response (VGPR) (HR = 0.12), and high-risk cytogenetic abnormalities (HRCA) (HR = 2.29) were all significant prognostic indicators of PFS. Similarly, HRCA (HR = 2.32), CRS (HR = 0.36), and a best response of ≥VGPR (HR = 0.16) were all independent prognostic factors of overall survival (OS). Of the 80 patients who received prior BDT, 22 received CAR-T (28%) and 58 (72%) received BsAbs. The median PFS was 6.3 months in the CAR-T group, with 64% ORR, vs. 4.4 months in the BsAb group, with 53% ORR (PFS HR, 0.93). Only the best response, ≥VGPR (HR = 0.20), achieved significance in the multivariable model. Conclusions: CAR-T led to more durable responses in the BDT-naïve setting than BsAbs. Responses to either modality were weaker in the BDT-exposed setting. These results highlight the importance of sequencing BDT in RRMM.

## Simple Summary

B-cell maturation antigen (BCMA)-directed therapies (BDT) are approved for the treatment of relapsed/refractory multiple myeloma (RRMM). Several treatment modalities exist including chimeric antigen receptor T-cell therapy (CAR-T), bispecific antibodies (BsAbs), and antibody-drug conjugates (ADC). The effectiveness of BCMA-targeting CAR-T and BsAbs has not been compared directly in clinical trial so real-world data provides insight into relative effectiveness. We present retrospectively-collected data comparing CAR-T and BsAbs in patients with RRMM from major US academic institutions. We also explored the impact of prior BDT (BDT-exposed, versus BDT-naïve). CAR-T led to more durable responses in the BDT-naïve setting than BsAbs. Responses to either modality were weaker in the BDT-exposed setting. Furthermore, our data suggest that the washout period and the choice of first BDT impact response rates in the exposed setting. These results highlight the importance of sequencing BDT in RRMM.

## 1. Introduction

Multiple myeloma (MM) is a plasma cell malignancy characterized by end-organ damage, including renal failure, hypercalcemia, anemia, and lytic bone lesions [1]. The current standard of care is a multi-agent induction regimen incorporating an anti-CD38 monoclonal antibody, a proteasome inhibitor (PI), an immunomodulatory drug (IMiD; typically lenalidomide), and corticosteroids, administered in triplet or quadruplet combinations, followed by autologous stem cell transplantation in transplant-eligible patients [2,3,4,5]. Overall survival in patients with standard-risk MM is >10 years, but high-risk patients relapse sooner, requiring subsequent lines of therapy [6]. With the expanding therapeutic armamentarium, including more than 25 approved agents and multiple cellular therapies as of 2026, optimal treatment selection in relapsed/refractory MM (RRMM) remains a major clinical challenge. Importantly, outcomes remain poor in heavily pretreated populations such as penta-refractory MM, where resistance to the five major drugs and three major drug classes is associated with a median overall survival of less than one year, underscoring the urgent need for novel therapeutic strategies [7].

B-cell maturation antigen (BCMA) is highly expressed in plasma cells from patients with newly diagnosed and RRMM, compared with normal marrow mononuclear cells [8]. Several BCMA-directed therapy (BDT) modalities have been approved by the FDA for RRMM, including chimeric antigen receptor T cell (CAR-T) therapies, bispecific antibodies (BsAbs), and antibody-drug conjugates (ADCs). Approved CAR-T cell therapies—idecabtagene vicleucel (ide-cel) and ciltacabtagene autoleucel (cilta-cel)—require leukapheresis, ex vivo T-cell engineering, lymphodepleting chemotherapy, and post-infusion monitoring [9,10]. In contrast, BCMA-directed BsAbs (e.g., teclistamab, elranatamab, and linvoseltamab) are off-the-shelf agents administered with step-up dosing followed by continuous treatment at regular intervals [11,12,13].

BCMA-targeted cellular and immune-based therapies have demonstrated deep and durable responses, including in higher-risk patient populations such as those with extramedullary disease (EMD), high-risk cytogenetic abnormalities (HRCAs), and plasma cell leukemia (PCL) [14,15,16,17]. To date, no randomized trials have directly compared the efficacy of different BCMA-directed modalities, and the optimal sequencing of these therapies remains undefined. Emerging evidence suggests that prior exposure to BCMA-directed therapy may adversely impact subsequent treatment efficacy, potentially through alterations in the tumor microenvironment, including reduced T-cell fitness, increased exhaustion, and impaired CAR-T expansion [18,19,20]. Given these considerations, determining the relative effectiveness of available BCMA-directed strategies—and the impact of prior exposure—is critical for optimizing treatment sequencing in RRMM.

In this multicenter retrospective analysis, we compared outcomes of patients treated with BCMA-directed CAR-T therapy versus BsAbs, stratified by prior BCMA exposure status.

## 2. Materials and Methods

### 2.1. Study Design and Setting

We conducted a retrospective, multicenter cohort study to evaluate the real-world efficacy of CAR-T therapy versus BsAbs in patients with RRMM. Patients were categorized into two cohorts based on their prior BCMA exposure status (BDT-naïve vs. BDT-exposed). Data were collected from seven participating U.S. academic centers between May 2021 and April 2024. Patients were identified using institutional electronic medical records (EMRs). The US Myeloma Innovations Research Collaborative (USMIRC) facilitated interinstitutional collaboration, study coordination, and manuscript development.

### 2.2. Patient Population

Patients were eligible if they were ≥18 years of age, had a diagnosis of RRMM according to the International Myeloma Working Group (IMWG) criteria [1], and had measurable disease. RRMM was defined as progression within 60 days of the completion of the most recent therapy or failure to respond to the most recent treatment, irrespective of the completion time. Patients with inadequate baseline or follow-up data were excluded from survival analysis. All eligible patients were included in this analysis.

Medical records were reviewed for baseline demographic and descriptive variables, response data, and adverse events of therapy. Treatment responses were determined according to the International Myeloma Work Group (IMWG) criteria [1]. Adverse events (AEs) were graded according to the Common Terminology Criteria for Adverse Events (CTCAE) v5.0 criteria. HRCAs were defined as the presence of genomic aberrations, including deletion (17p), t(4;14), t(14;16), at any time prior to the initiation of therapy.

### 2.3. Outcomes and Assessments

Primary outcomes included progression-free survival (PFS), defined as the time from treatment initiation to progression or death, and overall survival (OS), defined as the time from treatment initiation to death from any cause or to last follow-up. Secondary outcomes included response depth, prognostic factors, and adverse events. Acute infection was defined as a clinically or microbiologically confirmed infection within 30 days of treatment requiring antimicrobial therapy. Immune effector cell-associated neurotoxicity syndrome (ICANS) was analyzed both as a binary variable (presence/absence) and by maximum grade to distinguish occurrence from severity.

### 2.4. Statistical Analysis

Patient and disease characteristics were summarized using descriptive statistics. Continuous variables are described as mean ± SD, or median and interquartile range (IQR), while categorical variables are reported as frequencies and percentages. Between-group differences were assessed using Fisher’s exact test or the chi-square test for categorical variables. The Mann-Whitney U test was used to compare continuous variables. In the BDT-exposed cohort, outcome analyses were restricted to patients with evaluable data for each endpoint. Fifty-seven of the 58 teclistamab-treated patients were evaluable for overall response and overall survival, and 56 were evaluable for progression-free survival. Denominators are indicated in each table and figure legend.

OS and PFS were assessed using the Kaplan-Meier method, and differences between the survival curves were evaluated using the log-rank test. Univariate and multivariable Cox proportional hazards regression models were used to estimate the hazard ratios (HRs) and 95% confidence intervals (CIs). Variables significant in univariate analysis, together with clinically relevant covariates selected a priori as established prognostic factors in RRMM (age, extramedullary disease, and Revised International Staging System [R-ISS] stage), were entered into the multivariable models. Where variables were collinear, only the composite was retained. For example, individual cytogenetic lesions [del(17p), t(4;14), t(14;16)] were represented by the high-risk cytogenetics variable. Restricted Mean Survival Time (RMST) analysis was used to adjust for the uneven follow-up period. Because treatment allocation was not randomized, inverse probability of treatment weighting (IPTW) was used to address baseline imbalance in the BDT-naïve cohort. Propensity scores for receipt of CAR-T versus BsAb were estimated by multivariable logistic regression. Stabilized average treatment effect weights were calculated and trimmed at the 1st and 99th percentiles. Covariate balance was assessed by standardized mean differences (|SMD| < 0.10 considered balanced), and the effective sample size was calculated as (Σw)^2^/Σw^2^. IPTW-weighted Kaplan-Meier curves and weighted Cox proportional hazards models with robust variance estimators were fitted for PFS and OS, together with doubly robust models additionally including all propensity score covariates. Analyses were performed using the WeightIt (v2.0.0), cobalt (v4.6.3), survey (v4.5), and survival (v3.8.3) packages.

All analyses were conducted using R v4.4.2. All statistical tests were two-sided, and a *p*-value of ≤0.05 was considered significant [21]. Additional supporting analyses are provided in the Supplementary Materials (Figures S1–S4 and Tables S1–S7).

## 3. Results

### 3.1. BDT-Naïve Patients

#### 3.1.1. Patient Characteristics

From a total of 276 BDT-naïve patients included in the analysis, 169 (61%) received CAR-T treatment upfront, with 106 (63%) receiving ide-cel and 63 (37%) receiving cilta-cel (Table 1). All 107 patients in the BsAb group received teclistamab (100%). Median follow-up in the CAR-T and BsAb groups was 14.5 and 9.2 months, respectively. Patients in the CAR-T group were younger than those in the BsAb group (median age, 66 vs. 70 years; *p* < 0.001). Both the CAR-T and BsAb groups had comparable race (82% vs. 81% White, respectively) and sex (60% vs. 61% male, respectively) profiles. Patients receiving CAR-T had better performance status than those receiving BsAbs (Eastern Cooperative Oncology Group [ECOG] 0–1: 92% vs. 81%; *p* = 0.045). Among patients treated with CAR-T and BsAbs, 71% and 68% had R-ISS stage I–II disease, respectively (*p* = 0.7). Patients in the BsAb group had a greater prevalence of renal impairment, with a higher proportion of patients with creatinine clearance (CrCl) <45 mL/min than the CAR-T group (20% vs. 9.5%, *p* = 0.019).

Compared with the BsAb group, the CAR-T group had a higher median number of prior lines of therapy (6 vs. 4, *p* < 0.001) and a higher proportion of patients with a prior stem cell transplant (82% vs. 55%, *p* < 0.001). The proportions of triple-class refractory (*p* = 0.19) and penta-class refractory (*p* > 0.9) patients did not differ between groups. The CAR-T group had a higher proportion of patients with del(17p) (21% vs. 10%, *p* = 0.022) and a lower proportion of patients with t(14;16) (3.0% vs. 9.5%, *p* = 0.028) than the BsAb group. The proportion of patients with HRCA was similar between groups (30% CAR-T vs. 21% BsAb, *p* = 0.13).

#### 3.1.2. Adverse Events

A total of 269 and 209 BDT-naïve patients had available absolute neutrophil count (ANC) values on days 30 and 90, respectively. Patients who received CAR-T therapy had higher rates of grade ≥3 neutropenia on day 30 post-treatment than those who received teclistamab (grade 3–4 neutropenia: 44% vs. 10.9%, *p* < 0.001). This difference decreased at 90 days but was still statistically significant (grade 3–4 neutropenia: 12.5% vs. 9.5%, *p* = 0.027). Hemoglobin and platelet values were evaluated in 266 and 209 patients on days 30 and 90, respectively. Both treatments showed similar levels of anemia on day 30 post-treatment (grade 2–3 anemia: 44% CAR-T vs. 35% BsAbs, *p* = 0.5); however, CAR-T patients had increased rates of anemia on day 90 post-treatment (grade 2–3 anemia: 36.9% CAR-T vs. 18.7% BsAbs, *p* = 0.026). Platelet counts were significantly lower in patients treated with CAR-T cells on both day 30 (grade 3–4 thrombocytopenia: 45% vs. 11.9%, *p* < 0.001) and day 90 (grade 3–4 thrombocytopenia: 19.1% vs. 5.5%, *p* < 0.001) post-treatment. Patients on BsAbs had a higher rate of acute infection, with 43% developing an infection compared to 16% in the CAR-T cohort (*p* < 0.001). The incidence of cytokine release syndrome (CRS) was higher in patients who received CAR-T cell therapy (grade 2–3 CRS: 22.6% vs. 8.4%, *p* < 0.001). Lastly, the incidence of immune effector cell-associated neurotoxicity syndrome (ICANS) was not different between the groups (grade 3–4 ICANS: 3% CAR-T vs. 1.9% BsAb, *p* = 0.8).

### 3.2. Efficacy

#### 3.2.1. Response Rate

**Figure 1:**
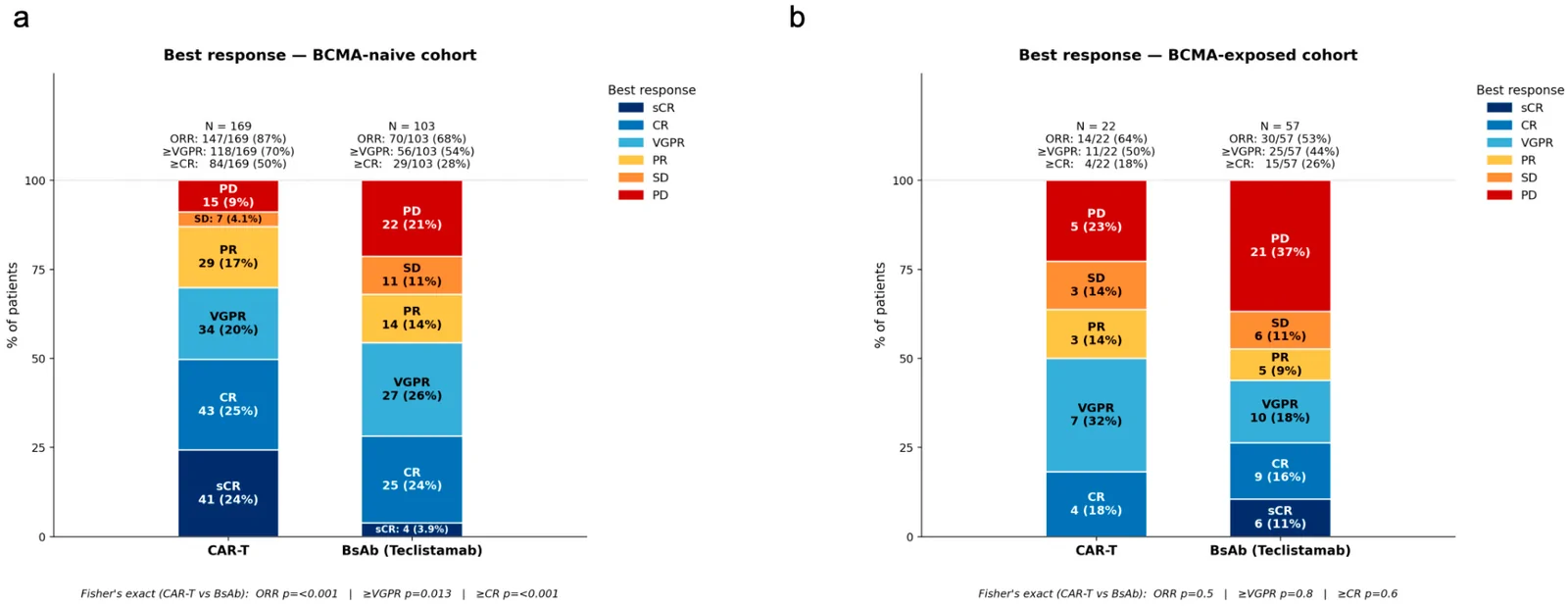
Overall response rates to CAR-T versus bispecific antibodies, stratified by prior BCMA exposure. Stacked bar plots showing best response distribution (stringent complete response [sCR], complete response [CR], very good partial response [VGPR], partial response [PR], stable disease [SD], progressive disease [PD]) to CAR T-cell therapy versus bispecific antibodies (BsAbs). (**a**) BDT-naïve cohort. (**b**) BDT-exposed cohort (CAR-T n = 22 vs. BsAb n = 57). Between-group differences were assessed using Fisher’s exact test. BDT, BCMA-directed therapy; sCR, stringent complete response; CR, complete response; VGPR, very good partial response; PR, partial response; MR, minimal response; SD, stable disease; PD, progressive disease.

ORR was 87% in the CAR-T group compared with 68% in the BsAb group. Deeper responses were observed in the CAR-T group, with 24% of patients achieving sCR compared with 3.9% in the BsAb group. In contrast, CR (25% vs. 24%), VGPR (20% vs. 26%), and PR (17% vs. 14%) rates were similar between the CAR-T and BsAb groups (Figure 1a).

#### 3.2.2. Progression-Free Survival

**Figure 2:**
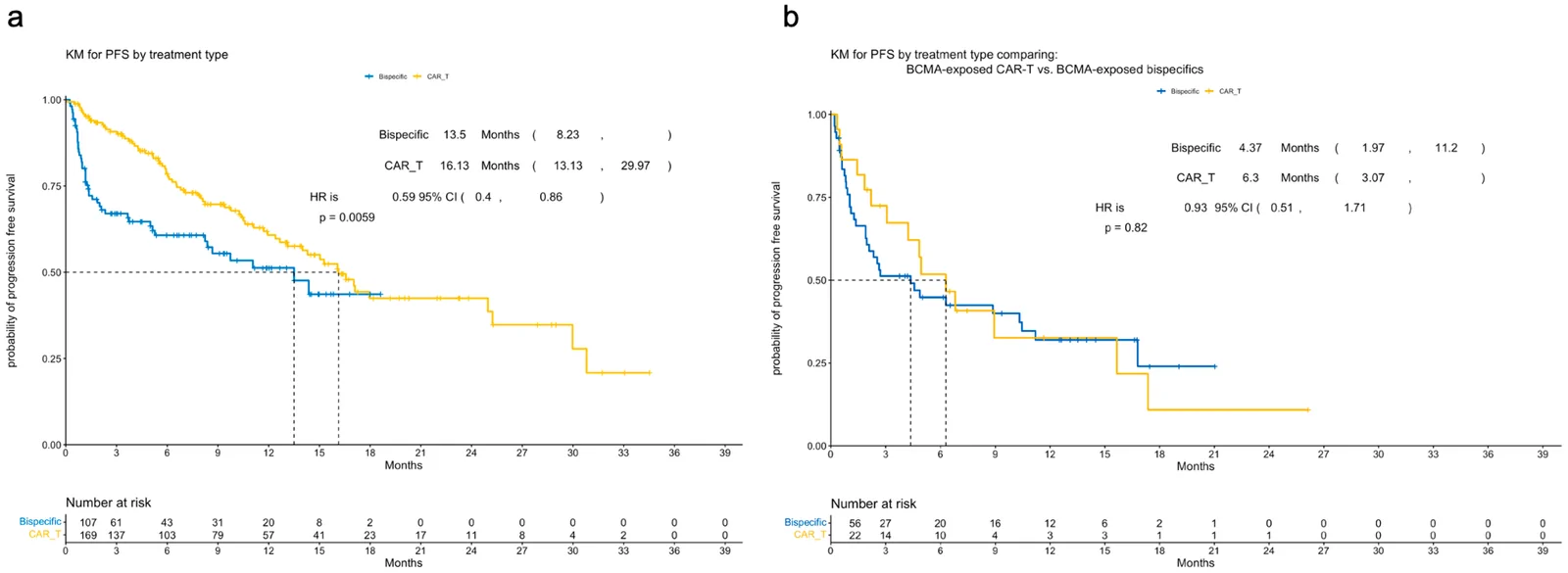
Progression-free survival in the BDT-naïve and BDT-exposed cohorts. Kaplan-Meier curves of progression-free survival (PFS) for patients receiving CAR T-cell therapy versus bispecific antibodies (**a**) BDT-naïve cohort. (**b**) BDT-exposed cohort. Differences between survival curves were tested using the log-rank test; hazard ratios (HR) with 95% confidence intervals were estimated using univariate Cox proportional-hazards regression.

Forty-one percent of patients in the CAR-T group and 43% in the BsAb group progressed on their respective therapies. The median PFS was significantly longer in the CAR-T group than in the BsAb group (16.1 vs. 13.5 months, *p* = 0.007; Tables S1 and S2, Figure 2a). In a subgroup analysis, median PFS was not reached with cilta-cel, and PFS was significantly shorter with BsAbs (BsAb vs. cilta-cel: HR = 4.04, 95% CI: 2.01–8.16, *p* < 0.001) (Figure S1a). A landmark analysis at day 30 after CAR-T infusion, performed to address immortal time bias, also favored CAR-T over BsAbs (BsAb vs. CAR-T: HR = 2.86, 95% CI: 1.31–6.26, *p* = 0.009) (Table S3).

On univariate analysis of the combined cohort, significant predictors of PFS were treatment type (CAR-T vs. BsAbs, HR = 0.59), presence of EMD (HR = 1.77), presence of HRCA (HR = 1.62), occurrence of CRS (HR = 0.63), best response of ≥VGPR (HR = 0.20), and lines of therapy (LOT; HR = 1.09). Multivariable Cox regression analysis revealed that receiving CAR-T (HR = 0.41, 95% CI: 0.26–0.66, *p* < 0.001), CRS of any grade (HR = 0.50, 95% CI: 0.33–0.76, *p* = 0.002), and achieving a best response of ≥VGPR (HR = 0.12, 95% CI: 0.08–0.19, *p* < 0.001) were all independent predictors of improved PFS. In contrast, high-risk cytogenetics (HR = 2.29, 95% CI: 1.50–3.50, *p* < 0.001) and increased LOT (HR = 1.23, 95% CI: 1.14–1.34, *p* < 0.001) were significant independent predictors of worse PFS (Table S1).

In the treatment-stratified univariate Cox regression, achieving a response of ≥VGPR was the only significant predictor of PFS in the BsAb group (HR = 0.06, 95% CI: 0.03–0.14, *p* < 0.001). In the CAR-T group, univariate and multivariable Cox regression analyses revealed that increased lines of therapy (HR = 1.2, 95% CI: 1.07–1.34, *p* = 0.002) and high-risk cytogenetics (HR = 2.47, 95% CI: 1.48–4.13, *p* < 0.001) were both independent poor prognostic factors, whereas a best response of ≥VGPR (HR = 0.20, 95% CI: 0.11–0.35, *p* < 0.001) was a good prognostic factor (Table S2).

#### 3.2.3. Overall Survival

**Figure 3:**
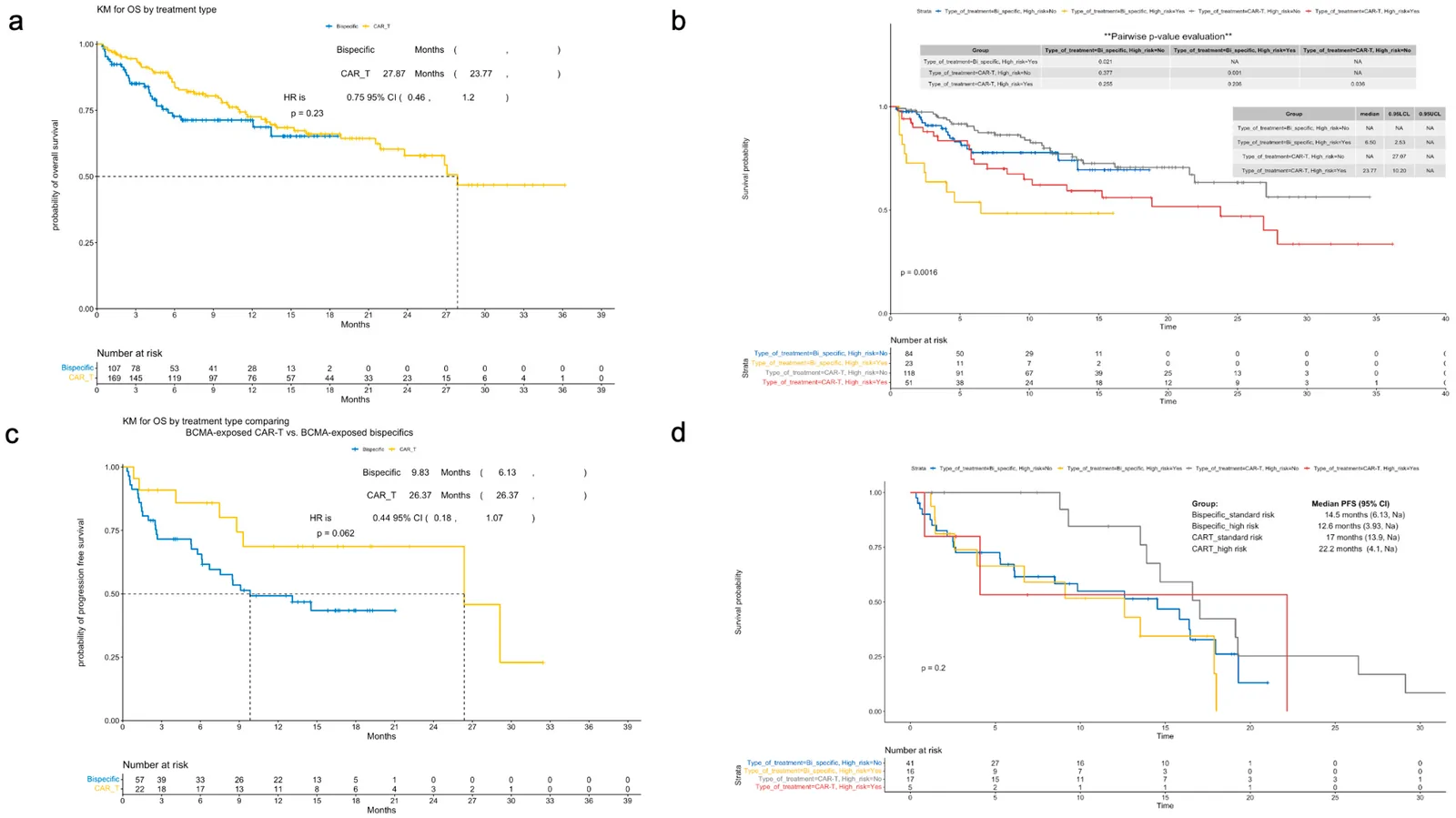
Overall survival in the BDT-naïve and BDT-exposed cohorts, with and without stratification by cytogenetic risk. Kaplan-Meier curves of overall survival (OS). (**a**) OS in BDT-naïve patients receiving CAR-T versus BsAbs. (**b**) OS in BDT-naïve patients stratified by treatment and cytogenetic risk (standard-risk cytogenetic abnormalities [SRCA]-CAR-T, SRCA-BsAb, HRCA-CAR-T, HRCA-BsAb); HRCA patients receiving BsAbs had the shortest median OS (6.5 months) while SRCA patients in both arms had the best outcomes (Median OS not reached [NA, not available] in both). (**c**) OS in BDT-exposed patients receiving CAR-T versus BsAbs (**d**) OS in BDT-exposed patients stratified by treatment and cytogenetic risk (overall log-rank *p* = 0.20).

Overall survival (OS) was not significantly different between the cohorts, with a median OS of 27.9 months in the CAR-T group, whereas median OS was not reached in the BsAb group owing to insufficient follow-up (HR = 0.75, 95% CI: 0.46–1.20, *p* = 0.2) (Table 2 and Table 3, Figure 3a). In the cilta-cel vs. BsAb subgroup analysis, median OS was not reached in either group; cilta-cel was associated with longer OS than BsAbs (BsAb vs. cilta-cel: HR = 2.30, 95% CI: 1.05–5.04, *p* = 0.038) (Figure S1b). A landmark analysis at day 30 after CAR-T infusion, performed to address immortal time bias, also favored CAR-T over BsAbs for OS (BsAb vs. CAR-T: HR = 2.48, 95% CI: 1.00–6.12, *p* = 0.049) (Table S3).

**Figure 4:**
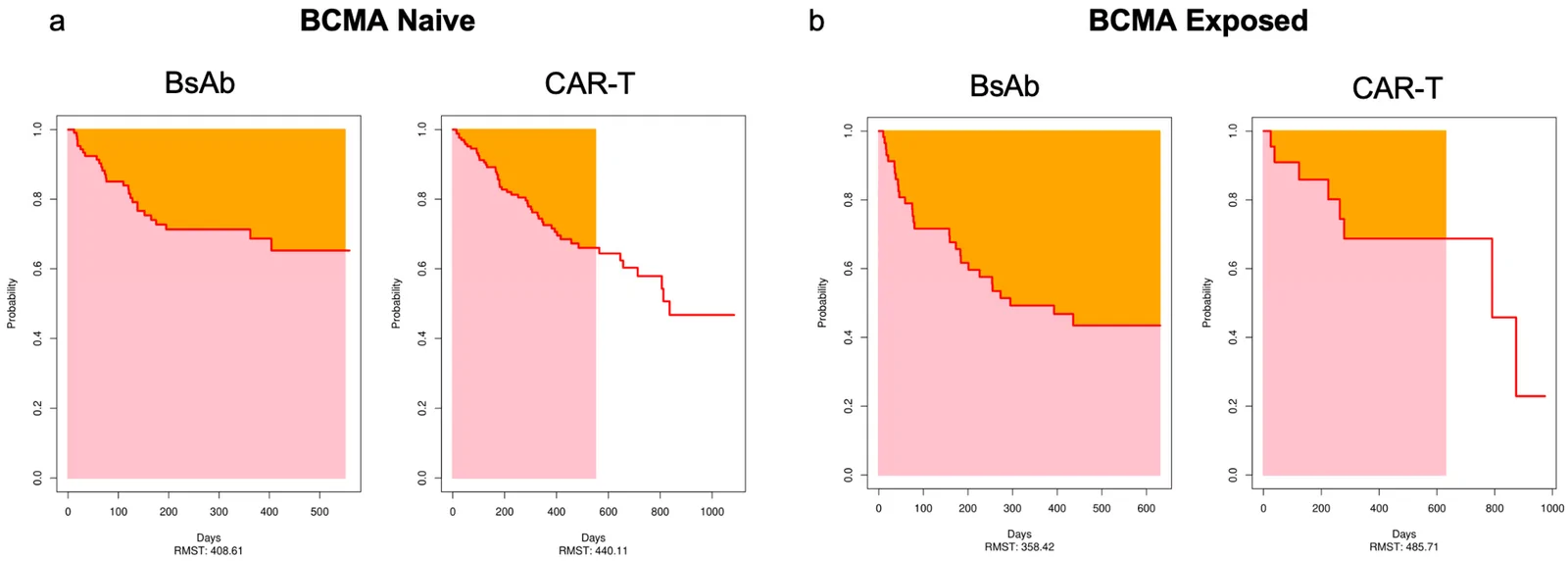
Restricted mean survival time (RMST) analysis of overall survival. RMST curves comparing CAR-T versus bispecific antibodies to address differences in follow-up duration between cohorts. (**a**) BDT-naïve cohort with a truncation time of 550 days (**b**) BDT-exposed cohort with a truncation time of 630 days.

To account for differences in follow-up between cohorts, we used the restricted mean survival time (RMST) method with a truncation time of 550 days. The RMST was 408.6 (SE = 22.4; 95% CI: 364.7–452.5) days in the BsAb arm and 440.1 (SE = 14.5; 95% CI: 411.8–468.4) days in the CAR-T arm. The RMST difference (BsAb–CAR-T) was −31.5 days (95% CI: −83.8 to 20.8; *p* = 0.24), indicating that, within the 550-day window, patients in the CAR-T arm lived on average 31.5 days longer than those in the BsAb arm, although this difference was not statistically significant (Table S4, Figure 4a). Risk stratification revealed that patients with standard-risk cytogenetics (SRCA) receiving CAR-T therapy (median OS not reached [NR]) and those with SRCA receiving BsAbs (median OS: NR) had the best OS. Patients with HRCA receiving CAR-T therapy (median OS = 23.8 months) and those with HRCA receiving BsAbs (median OS = 6.5 months) had the worst OS (Figure 3b).

Similar to PFS, HRCA (HR = 2.32, 95% CI: 1.42–3.79, *p* < 0.001), CRS (HR = 0.36, 95% CI: 0.22–0.61, *p* < 0.001), and a best response of ≥VGPR (HR = 0.16, 95% CI: 0.09–0.26, *p* < 0.001) remained independent predictors of OS on multivariable analysis (Table 2). In the treatment-stratified analysis, independent predictors of OS in the CAR-T group were HRCA (HR = 1.89, 95% CI: 1.03–3.45, *p* = 0.043) and a best response of ≥VGPR (HR = 0.29, 95% CI: 0.16–0.52, *p* < 0.001). In the BsAb group, in addition to HRCA (HR = 3.85, 95% CI: 1.60–9.26, *p* = 0.003) and a best response of ≥VGPR (HR = 0.06, 95% CI: 0.02–0.20, *p* < 0.001), other factors associated with prognosis included any ICANS event (HR = 3.02, 95% CI: 1.09–8.35, *p* = 0.043) and CRS (HR = 0.21, 95% CI: 0.08–0.55, *p* < 0.001) (Table 3).

#### 3.2.4. Inverse Probability of Treatment Weighting (IPTW)

In light of the baseline differences between the BsAb and CAR-T cohorts, we fitted IPTW and doubly robust (DR) models on PFS and OS to account for these differences. The models adjusted for age, ECOG performance status, LOT, prior autologous stem cell transplantation (ASCT), CrCl < 45 mL/min, EMD, and HRCA. Seventeen CAR-T patients were excluded from the analysis due to missing ECOG status. After adjustment, all covariates achieved an absolute SMD <0.1, except EMD (SMD = 0.103) and age (SMD = −0.167). The largest baseline difference was in LOT, which was well balanced after weighting (SMD 0.687 before vs. 0.033 after) (Table S7). In terms of PFS, the IPTW and DR models showed that CAR-T was associated with better PFS than BsAbs (IPTW HR: 0.43, 95% CI: 0.27–0.67, *p* < 0.001; DR HR: 0.33, 95% CI: 0.20–0.55, *p* < 0.001). For OS, the IPTW model showed a trend favoring CAR-T, and the DR model showed a significant benefit (IPTW HR: 0.59, 95% CI: 0.34–1.04, *p* = 0.066; DR HR: 0.49, 95% CI: 0.27–0.89, *p* = 0.019) (Figure S4).

### 3.3. BDT-Exposed Patients

#### 3.3.1. Patient Characteristics

Of the 80 patients who received prior BDT, 58 (72.5%) received teclistamab and 22 (27.5%) received CAR-T (cilta-cel, n = 10; ide-cel, n = 12) (Table 4). The CAR-T and BsAb groups had similar median ages (64 vs. 68 years, respectively). Race (73% vs. 79% White), sex (50% vs. 59% male), and ECOG performance status (ECOG 0–1: 80% vs. 83%) were also similar. HRCA profiles did not differ significantly between the two groups (23% in CAR-T vs. 28% in BsAb; *p* = 0.8). Median LOT was 7.5 in the CAR-T group and 8 in the BsAb group (*p* = 0.6), but more patients had a prior transplant in the CAR-T group (100%) compared with the BsAb group (78%; *p* = 0.015). Triple-class refractory (*p* = 0.13) and penta-class refractory (*p* = 0.3) status was comparable between the groups. Among patients treated with CAR-T and BsAbs, approximately 78% and 74% had R-ISS stage I–II disease, respectively (*p* = 0.6). Prior BCMA regimens differed significantly between the two cohorts (*p* < 0.001). In the BsAb cohort (n = 58), 29 (50%) patients had previous exposure to CAR-T therapy, 12 (20.7%) had received the ADC belantamab mafodotin, 6 (10.3%) had received prior bispecific antibodies, and 11 (19%) received a combination of the above. In contrast, most patients in the CAR-T group received belantamab mafodotin (n = 12, 54.5%) or bispecific antibodies (n = 9, 41%); only one patient received agents from both classes.

#### 3.3.2. Adverse Events

Hematologic adverse events occurred in both groups. On day 30 post-treatment, ANC values were missing for 8 BsAb patients, and hemoglobin and platelet values were missing for 9 patients (8 BsAb and 1 CAR-T). A total of 3 CAR-T and 23 BsAb patients were missing all three variables on day 90 post-treatment. Patients receiving CAR-T therapy had higher rates of neutropenia on day 30 (grade 3–4 neutropenia: 41% vs. 14%, *p* < 0.001), a difference that was no longer significant on day 90 post-treatment (grade 3–4 neutropenia: 5.3% vs. 5.8%, *p* = 0.7). Anemia was also more frequent with CAR-T on day 30 (grade 2–3 anemia: 57% vs. 30%, *p* = 0.048), with a larger difference on day 90 (grade 2–3 anemia: 42% vs. 14.3%, *p* = 0.003) post-treatment. CAR-T was also associated with more severe thrombocytopenia on day 30 (grade 3–4 thrombocytopenia: 52.5% vs. 10%, *p* = 0.002) and day 90 (grade 3–4 thrombocytopenia: 27% vs. 0%, *p* = 0.030) post-treatment. Patients receiving BsAbs had higher rates of acute infection than those receiving CAR-T (48% vs. 14%, *p* = 0.005). CAR-T patients had similar rates of CRS to BsAb patients (grade 1–2 CRS: 69% CAR-T vs. 51.4% BsAbs, *p* = 0.13). Finally, the incidence of ICANS was similar in both groups (grade 3–4 ICANS: 0% CAR-T vs. 3.4% BsAbs, *p* > 0.9) (Table 4).

### 3.4. Efficacy

#### 3.4.1. Response Rate

ORR was 64% in the CAR-T group compared with 53% in the BsAb group. Patients treated with CAR-T and BsAbs achieved sCR in 0% vs. 11% of cases, CR in 18% vs. 16% of cases, VGPR in 32% vs. 18% of cases, and PR in 14% vs. 8.8% of cases (Figure 1b). CAR-T patients were hospitalized for a median of 11.0 days (IQR: 9.0–16.0), while BsAb patients were hospitalized for a median of 8.0 days (IQR: 7.0–11.0; *p* = 0.018). Stratification of ORR by washout period and prior BCMA exposure showed that patients who received a different BCMA-directed modality >6 months after their previous one had the best responses (previous CAR-T to teclistamab: ORR = 65%; previous ADC to CAR-T: ORR = 89%). Patients who had previously received BsAbs had poorer responses, regardless of the second BCMA modality (Figure S2).

#### 3.4.2. Progression-Free Survival

Disease progression occurred in 68% of the CAR-T group and 60% of the BsAb group (Table 4). The median PFS was poor in both groups: 6.3 months in the CAR-T group vs. 4.4 months in the BsAb group (HR = 0.93, *p* = 0.82) (Figure 2b). Age and best response of ≥VGPR were the only two significant predictors of PFS in the univariate analysis, with only the best response of ≥VGPR (HR = 0.20, 95% CI: 0.11–0.39, *p* < 0.001) remaining significant in the multivariable model. In the BsAb cohort, a best response of ≥VGPR was the only predictor of PFS (HR = 0.14, 95% CI: 0.06–0.33, *p* < 0.001), whereas age (HR = 0.90, 95% CI: 0.84–0.97, *p* = 0.006) was the only significant predictor of PFS in the CAR-T group in the multivariable model (Tables S5 and S6). Subgroup analysis showed that there was no difference in PFS between cilta-cel and BsAbs (HR = 1.91, 95% CI: 0.66–5.48, log-rank *p* = 0.22) (Figure S3a). A landmark analysis at day 30 showed no significant difference in PFS between groups (*p* = 0.87) (Table S3).

#### 3.4.3. Overall Survival

At the time of data collection, median follow-up was 16.4 and 15.6 months for the CAR-T and BsAb groups, respectively. The median OS in patients receiving CAR-T was 26.4 months, compared with 13.1 months in the BsAb group, with no statistically significant difference (HR = 0.44, 95% CI: 0.18–1.07, *p* = 0.062) (Figure 3c). Further stratification using the cytogenetic profile and treatment type did not show a significant difference in OS (*p* = 0.20), with HRCA and SRCA patients receiving BsAbs and HRCA patients receiving CAR-T showing substantial overlap in the Kaplan–Meier curves (Figure 3d). Given the short follow-up period, the RMST analysis used a truncation time of 630 days (the furthest time point at which both cohorts had adequate follow-up). RMST was 358.4 days (SE = 35.4; 95% CI: 289.0–427.8) in the BsAb cohort. In the CAR-T cohort, the RMST was longer, at 485.7 days (SE = 49.9; 95% CI: 387.8–583.6). The RMST difference (BsAb–CAR-T) was statistically significant, indicating that patients in the CAR-T group, on average, lived 127 days longer within the 630-day window (RMST difference = −127.29 days, 95% CI: −247.29 to −7.29, *p* = 0.038) (Table S4, Figure 4b). Multivariable Cox regression revealed that a best response of ≥VGPR was the only significant predictor of OS in the combined cohort (HR = 0.20, 95% CI: 0.09–0.45, *p* < 0.001) (Table 5). In the BsAb group, EMD (HR = 3.21, 95% CI: 1.36–7.55; *p* = 0.007), maximum ICANS grade (HR = 0.11, 95% CI: 0.02–0.58; *p* = 0.016), and best response of ≥VGPR (HR = 0.11, 95% CI: 0.04–0.31; *p* < 0.001) were independent predictors of OS. In contrast, the only significant predictor of OS in the CAR-T cohort was t(14;16) (HR = 15.7, 95% CI: 1.42–175, *p* = 0.025) (Table 6).

Cilta-cel was associated with longer OS than BsAbs (log-rank *p* = 0.012; the HR was not estimable because no deaths occurred in the cilta-cel group) (Figure S3b). A landmark analysis at day 30 showed no significant difference in OS between cilta-cel and BsAbs (*p* > 0.9) (Table S3).

## 4. Discussion

To our knowledge, this represents one of the largest real-world multicenter retrospective cohorts evaluating BDT modalities in both BDT-naïve and BDT-exposed settings. Treatment assignment was non-randomized, and patients receiving CAR-T in the BDT-naïve setting were generally younger, more heavily pretreated, had better performance status, and had less renal impairment. These differences were addressed with the IPTW and DR models. In contrast, baseline characteristics were more balanced between treatment groups in the BDT-exposed setting. Referral bias and access limitations likely contributed to confounding, particularly because only 20–30% of eligible patients ultimately receive CAR-T therapy [22]. Additionally, prior BCMA exposure in the BDT-exposed cohort was heterogeneous, with belantamab mafodotin exposure occurring more frequently in the CAR-T group than in the BsAb group (54.5% vs. 20.7%). These baseline differences may have influenced outcomes despite multivariable adjustment.

Both CAR-T and BsAbs demonstrated improved efficacy in the BDT-naïve setting compared with the BDT-exposed setting (ORR: CAR-T 87% vs. 64%; BsAbs 68% vs. 53%). A prior systematic review of 18 CAR-T and 7 BsAb studies showed higher ORR (92% vs. 60%, *p* < 0.001) and CR or better rates (57% vs. 32%, *p* < 0.001) with CAR-T [23], consistent with previous reports [17,24], although such comparisons should be interpreted cautiously due to baseline differences.

Our findings further align with evidence suggesting superior durability of response in BDT-naïve patients. Inferior outcomes in BDT-exposed patients likely reflect biologically resistant disease, potentially related to antigen modulation, T-cell exhaustion, and an immunosuppressive marrow microenvironment following prior BDT [18,19]. Future studies should stratify outcomes by the specific type of prior BDT rather than treating BCMA exposure as a single category.

In a national French real-world cohort of 154 patients relapsing after ide-cel, salvage anti-BCMA bispecific antibodies achieved a median PFS of only 3.8 months, compared with 9.2 months for bispecific antibodies directed at non-BCMA targets [25]. In our study, PFS with CAR-T was 16.1 months in BDT-naïve patients compared with 6.3 months in BDT-exposed patients likely reflecting the more advanced nature of disease in the BDT-exposed cohort. Subgroup analysis similarly showed stronger PFS and OS signals with cilta-cel. A meta-analysis of 1269 patients also found higher CR rates with CAR-T than BsAbs (54% vs. 35%, *p* < 0.01), although at the expense of increased toxicities, particularly CRS [24]. In another real-world analysis comparing CAR-T products with teclistamab, CAR-T therapy was associated with lower all-cause mortality, especially with cilta-cel [26]. Collectively, these findings are consistent with deeper and more durable responses with cilta-cel compared with ide-cel [27,28].

In the BDT-naïve cohort, overall survival numerically favored CAR-T, although the differences did not consistently reach statistical significance. The doubly robust model showed a significant OS difference favoring CAR-T (HR = 0.49, 95% CI: 0.27–0.89). The full cohort analysis showed a similar trend but did not reach statistical significance. This should be interpreted with caution, as the OS difference may partly reflect poorer outcomes with subsequent BDT after teclistamab than after CAR-T. HRCA remained a major adverse prognostic factor across cohorts. Prior studies from CARTITUDE-1 [29] and real-world ide-cel analyses similarly demonstrated inferior outcomes in HRCA patients [30], whereas CARTITUDE-4 showed that earlier use of cilta-cel may overcome some of this risk [31]. Our population had a median of ≥4 prior LOT, which may explain these differences and highlights the importance of earlier intervention.

Current indications are evolving, with cilta-cel and ide-cel approved after one and two prior LOT, respectively, while BsAbs have largely remained later-line therapies [32]. The optimal sequencing of BDT remains uncertain, and emerging evidence suggests reduced efficacy with sequential BCMA targeting, supporting alternative targets such as G protein-coupled receptor class C group 5 member D (GPRC5D) and Fc receptor homolog 5 (FcRH5) [33]. Earlier use of cilta-cel has been associated with improved T-cell fitness and longer PFS [34], with long-term outcomes from CARTITUDE studies suggesting durable disease control and possible functional cure in a subset of patients [29,35].

In our analysis, response rates appeared to improve when a different BCMA modality was administered following an adequate washout period, with favorable responses seen with CAR-T after ADC and with BsAbs after CAR-T (89% and 65%, respectively). These findings suggest that CAR-T may be preferred for fit, high-risk patients given its deeper and more durable responses, whereas BsAbs may provide a more practical option for patients requiring rapid treatment initiation or those with limited CAR-T eligibility or access [36,37]. Additionally, fixed-duration or dose-spaced teclistamab approaches may reduce treatment burden and possibly improve the efficacy of subsequent BDT by limiting BCMA-specific resistance; in our cohort, patients who received any BDT after prior BsAbs had poorer responses than those previously exposed to other modalities. It is important to note, however, that infection risk remains substantial even after BsAb discontinuation [38,39].

Regarding safety, CAR-T was associated with more cytopenias, whereas BsAbs were associated with higher infection rates. Early cytopenias with CAR-T are usually the result of lymphodepleting therapy, while delayed-onset or persistent cytopenias are often multifactorial and may reflect the inflammatory effects of CAR T-cell therapy, baseline marrow reserve, prior treatment exposure, and infection. Prior studies similarly showed lower infection rates with CAR-T compared with BsAbs [10,26,27,40,41,42,43,44,45,46]. Continuous BsAb exposure may contribute to persistent immunosuppression and infection risk, emphasizing the need for standardized prophylaxis and supportive care strategies.

Despite its strengths, several limitations should be acknowledged. The retrospective design limits causal inference, and we lacked information on the time spent on prior BDT, outcomes on prior BDT, measurable residual disease (MRD) assessments, standardized response timing, bridging strategies, and uniform supportive care practices. In addition, our cohorts had significant baseline differences: CAR-T patients were younger, had better ECOG performance status, and were more heavily pretreated, whereas patients receiving BsAbs had more renal impairment. This was addressed by using IPTW and doubly robust models, but residual confounding due to incompletely balanced variables (EMD and age) persists. Additionally, our BsAb cohort consisted only of teclistamab, limiting generalizability to newer agents such as elranatamab and linvoseltamab [47]. The small sample size of our BDT-exposed cohort further limits the generalizability and robustness of our analysis. Prior studies suggest that longer intervals from prior BDT exposure and target switching strategies may improve outcomes [48]. Moreover, evolving IMWG high-risk definitions and lack of bridging treatment data may have further influenced results [49,50,51,52]. Differences in follow-up between cohorts also limit the interpretation of survival results. Lastly, institutional practices with prophylactic intravenous immunoglobulin (IVIG) and lymphodepletion may have affected certain outcomes, such as the incidence of infections and cytopenias.

## 5. Conclusions

In conclusion, this large real-world multicenter study demonstrates that CAR-T therapy is associated with deeper responses and longer progression-free survival in BDT-naïve patients, while both CAR-T and bispecific antibodies show limited durability following prior BCMA exposure. These findings highlight the importance of early integration of cellular therapies and underscore the need for optimized sequencing strategies, including the incorporation of alternative targets in RRMM. Prospective studies are needed to better define treatment selection and sequencing in this rapidly evolving therapeutic landscape.

## Figures and Tables

**Table 1:** Baseline demographic, clinical, safety outcomes, and disease characteristics of BDT-naïve patients receiving CAR T-cell therapy versus bispecific antibodies.

Characteristic	CAR-T N = 169 ^1^	BsAbs N = 107 ^1^	*p*-Value ^2^
**Age**	66 (60, 70)	70 (64, 76)	**<0.001**
**Race**			0.7
African American	24 (14%)	18 (17%)	
Asian	2 (1.2%)	1 (0.9%)	
Hispanic	1 (0.6%)	1 (0.9%)	
Other	3 (1.8%)	0 (0%)	
White	139 (82%)	87 (81%)	
**Sex**			>0.9
Female	68 (40%)	42 (39%)	
Male	101 (60%)	65 (61%)	
**ECOG**			**0.045**
0	23 (15%)	13 (12%)	
1	117 (77%)	74 (69%)	
2	12 (7.9%)	18 (17%)	
3	0 (0%)	2 (1.9%)	
Unknown	17	0	
**Heavy Isotype**			0.48
IGA	31 (18%)	22 (21%)	
IGD	0 (0%)	1 (0.9%)	
IGG	103 (61%)	60 (56%)	
IGM	0 (0%)	1 (0.9%)	
Light Chain Disease	35 (21%)	21 (19.1%)	
Unknown	0 (0%)	2 (1.9%)	
**Light Chain**			0.3
Kappa	105 (62%)	64 (60%)	
Lambda	64 (38%)	41 (38%)	
NA	0 (0%)	2 (1.9%)	
**R-ISS stage**			0.7
1	29 (22%)	23 (25%)	
2	65 (49%)	40 (43%)	
3	38 (29%)	29 (32%)	
Unknown	37	15	
**PCL**			0.4
No	166 (98%)	103 (96%)	
Yes	3 (1.8%)	4 (3.7%)	
**Amyloid**			0.7
No	165 (98%)	106 (99%)	
Yes	4 (2.4%)	1 (0.9%)	
**EF < 45%**			>0.9
No	130 (98%)	105 (98%)	
Yes	3 (2.3%)	2 (1.9%)	
Unknown	36	0	
**EMD**			0.13
No	118 (70%)	84 (79%)	
Yes	51 (30%)	23 (21%)	
**Prior SCT**			**<0.001**
No	31 (18%)	48 (45%)	
Yes	138 (82%)	59 (55%)	
**LOT**	6.00 (4.00, 8.00)	4.00 (4.00, 6.00)	**<0.001**
**Deletion 17p**			**0.022**
No	133 (79%)	94 (90%)	
Yes	36 (21%)	11 (10%)	
Unknown	0	2	
**t(4;14)**			0.2
No	154 (91%)	100 (95%)	
yes	15 (8.9%)	5 (4.8%)	
Unknown	0	2	
**t(14;16)**			**0.028**
No	164 (97%)	95 (90%)	
Yes	5 (3.0%)	10 (9.5%)	
Unknown	0	2	
**Cytogenetic risk group**			0.13
High-risk	51 (30%)	23 (21%)	
Standard-risk	118 (70%)	84 (79%)	
**Bortezomib**			0.4
Exposed	75 (44%)	41 (38%)	
NOT Exposed	2 (1.2%)	3 (2.8%)	
Refractory	92 (54%)	63 (59%)	
**Carfilzomib**			**0.001**
Exposed	43 (25%)	10 (9.3%)	
NOT Exposed	15 (8.9%)	16 (15%)	
Refractory	111 (66%)	81 (76%)	
**Lenalidomide**			0.14
Exposed	44 (26%)	18 (17%)	
NOT Exposed	2 (1.2%)	3 (2.8%)	
Refractory	123 (73%)	85 (80%)	
Unknown	0	1	
**Pomalidomide**			**<0.001**
Exposed	36 (21%)	9 (8.4%)	
NOT Exposed	14 (8.3%)	23 (21%)	
Refractory	119 (70%)	75 (70%)	
**anti-CD38**			0.9
Exposed	12 (7.1%)	6 (5.6%)	
NoT Exposed	3 (1.8%)	1 (0.9%)	
Refractory	154 (91%)	100 (93%)	
**Triple-class refractory**	123 (73%)	86 (80%)	0.19
**Penta-class refractory**	44 (26%)	28 (26%)	>0.9
**Product**			**<0.001**
Cilta-cel	63 (37%)	0 (0%)	
Ide-cel	106 (63%)	0 (0%)	
Teclistamab	0 (0%)	107 (100%)	
**CrCl < 45**			**0.019**
0	153 (91%)	86 (80%)	
1	16 (9.5%)	21 (20%)	
**CRS**			**<0.001**
0	31 (18%)	53 (50%)	
1	99 (59%)	45 (42%)	
2	38 (22%)	6 (5.6%)	
3	1 (0.6%)	3 (2.8%)	
**Hospitalization (days)**	10.0 (7.0, 15.0)	7.0 (6.0, 9.0)	**<0.001**
**Acute Infection**	27 (16%)	46 (43%)	**<0.001**
Unknown	0	1	
**Best Response**			**<0.001**
sCR	41 (24%)	4 (3.9%)	
CR	43 (25%)	25 (24%)	
VGPR	34 (20%)	27 (26%)	
PR	29 (17%)	14 (14%)	
SD	7 (4.2%)	11 (11%)	
PD	15 (8.9%)	22 (21%)	
Unknown	0	4	
**Progression**	69 (41%)	46 (43%)	0.7
**PFS**	240 (117, 441)	119 (35, 292)	**<0.001**
**Death**	51 (30%)	28 (26%)	0.5
**OS**	327 (164, 546)	179 (80, 362)	**<0.001**
**Median follow-up** (months)	14.5 (8.0, 23.0)	9.2 (4.4, 14.2)	—
**ANC Day 30**			**<0.001**
0	54 (32%)	75 (74%)	
1	12 (7.1%)	7 (6.9%)	
2	28 (17%)	8 (7.9%)	
3	51 (30%)	6 (5.9%)	
4	23 (14%)	5 (5.0%)	
Unknown	1	6	
**HgB Day 30**			0.5
0	43 (26%)	33 (33%)	
1	50 (30%)	33 (33%)	
2	49 (30%)	25 (25%)	
3	23 (14%)	10 (9.9%)	
Unknown	4	6	
**PLT Day 30**			**<0.001**
0	24 (15%)	53 (52%)	
1	45 (27%)	22 (22%)	
2	22 (13%)	14 (14%)	
3	39 (24%)	9 (8.9%)	
4	35 (21%)	3 (3.0%)	
Unknown	4	6	
**ANC Day 90**			**0.027**
0	87 (64%)	61 (82%)	
1	11 (8.1%)	4 (5.4%)	
2	20 (15%)	2 (2.7%)	
3	11 (8.1%)	3 (4.1%)	
4	6 (4.4%)	4 (5.4%)	
Unknown	34	33	
**HgB Day 90**			**0.026**
0	63 (47%)	39 (53%)	
1	22 (16%)	21 (28%)	
2	38 (28%)	12 (16%)	
3	12 (8.9%)	2 (2.7%)	
Unknown	34	33	
**PLT Day 90**			**<0.001**
0	45 (33%)	50 (68%)	
1	50 (37%)	17 (23%)	
2	14 (10%)	3 (4.1%)	
3	15 (11%)	3 (4.1%)	
4	11 (8.1%)	1 (1.4%)	
Unknown	34	33	
**ICANS**			0.8
0	132 (78%)	91 (85%)	
1	19 (11%)	9 (8.4%)	
2	12 (7.1%)	5 (4.7%)	
3	3 (1.8%)	2 (1.9%)	
4	2 (1.2%)	0 (0%)	
5	1 (0.6%)	0 (0%)	
**Stem Cell Boost**	11 (6.5%)	0 (0%)	NA
Unknown	0	107	

^1^ Median (Q1, Q3); n (%). ^2^ Mann-Whitney U test; Fisher’s Exact Test for Count Data with simulated *p*-value (based on 2000 replicates); Fisher’s exact test. Continuous variables are reported as median (interquartile range) and were compared using the Mann-Whitney U test; categorical variables are reported as n (%) and were compared using Fisher’s exact test or the chi-square test as appropriate. *p*-values < 0.05 are considered statistically significant and shown in bold. High-risk cytogenetic abnormalities (HRCAs) were defined as del(17p), t(4;14), or t(14;16) detected at any time prior to therapy initiation. Triple-class refractory: refractory to a proteasome inhibitor, an immunomodulatory drug, and an anti-CD38 monoclonal antibody. Penta-class refractory: refractory to ≥2 proteasome inhibitors, ≥2 immunomodulatory drugs, and an anti-CD38 monoclonal antibody. BDT, BCMA-directed therapy; BsAb, bispecific antibody; ECOG, Eastern Cooperative Oncology Group performance status; R-ISS, Revised International Staging System; CrCl, creatinine clearance; LOT, lines of therapy; NA, not available; SCT, stem cell transplant; EMD, extramedullary disease.

**Table 2:** Univariate and multivariable Cox analysis of overall survival in the combined BDT-naïve cohort.

	Univariate			Multivariable		
Characteristic	N	HR (95% CI)	*p*-Value	N	HR (95% CI)	*p*-Value
**Type of treatment**			0.2	276		0.8
Bispecific	107	—			—	
CAR-T	169	0.75 (0.46–1.20)			0.94 (0.55–1.61)	
**EMD**			0.2	276		0.6
No	202	—			—	
Yes	74	1.34 (0.84–2.15)			1.17 (0.71–1.92)	
**CrCl < 45**			0.11			
No	239	—				
Yes	37	1.64 (0.92–2.93)				
**R-ISS stage**			0.4	276		0.2
1	52	—			—	
2	105	1.5 (0.72–3.14)			2.09 (0.98–4.45)	
3	67	1.72 (0.80–3.71)			2 (0.87–4.59)	
unknown	52	1.15 (0.49–2.70)			1.53 (0.65–3.63)	
**ECOG (0–1 vs. 2–3)**	259	0.66 (0.36–1.23)	0.2			
**Prior transplants**			0.2			
No	79	—				
Yes	197	0.7 (0.43–1.13)				
**LOT**	276	1.09 (1.0–1.19)	0.072			
**Deletion 17p**			**0.028**			
No	229	—				
Yes	47	1.8 (1.09–2.97)				
**t(4;14)**			0.14			
No	256	—				
Yes	20	1.71 (0.88–3.32)				
**t(14;16)**			0.8			
No	261	—				
Yes	15	1.1 (0.44–2.71)				
**Age**	276	0.99 (0.97–1.02)	0.5	276	0.98 (0.95–1.00)	0.088
**High risk**			**0.002**	276		**<0.001**
No	202	—			—	
Yes	74	2.08 (1.33–3.25)			2.32 (1.42–3.79)	
**EF < 45%**			0.2			
No	271	—				
Yes	5	2.23 (0.70–7.09)				
**CRS**			**0.009**	276		**<0.001**
No	84	—			—	
Yes	192	0.54 (0.34–0.84)			0.36 (0.22–0.61)	
**Max CRS**	276	0.93 (0.53–1.63)	0.8			
**ICANS**			**0.008**	276		0.077
No	226	—			—	
Yes	50	2.07 (1.25–3.42)			1.74 (0.95–3.18)	
**Max ICANS**	276	0.35 (0.20–0.63)	**0.002**			
**At Least VGPR**	276	0.19 (0.12–0.31)	**<0.001**	276	0.16 (0.09–0.26)	**<0.001**

Abbreviations: CI = Confidence Interval, HR = Hazard Ratio.

**Table 3:** Treatment-stratified univariate and multivariable Cox analysis of overall survival in BDT-naïve patients.

	Bispecific, N = 107					CAR-T, N = 169				
	Univariate			Multivariable		Univariate			Multivariable	
Characteristic	N	HR (95% CI)	*p*-Value	HR (95% CI)	*p*-Value	N	HR (95% CI)	*p*-Value	HR (95% CI)	*p*-Value
**EMD**										
No	84	—				118	—			
Yes	23	1.28 (0.54–3.02)	0.6			51	1.44 (0.81–2.54)	0.2		
**CrCl < 45**										
No	86	—		—		153	—			
Yes	21	2.46 (1.07–5.65)	**0.033**	2.57 (0.94–7.02)	0.076	16	1.08 (0.46–2.53)	0.9		
**R-ISS stage**										
1	23	—				29	—			
2	40	0.96 (0.29–3.19)	>0.9			65	1.89 (0.72–4.93)	0.2		
3	29	2.37 (0.76–7.46)	0.14			38	1.43 (0.51–4.02)	0.5		
unknown	15	1.5 (0.40–5.58)	0.5			37	1.08 (0.35–3.31)	0.9		
**ECOG (0–1 vs. 2–3)**	107	0.59 (0.26–1.35)	0.2			152	0.94 (0.34–2.63)	>0.9		
**Prior transplants**										
No	48	—				31	—			
Yes	59	0.85 (0.40–1.78)	0.7			138	0.66 (0.34–1.30)	0.2		
**LOT**	107	1.14 (0.98–1.32)	0.1			169	1.1 (0.98–1.23)	0.1		
**Deletion 17p**										
No	96	—				133	—			
Yes	11	1.77 (0.67–4.66)	0.2			36	1.96 (1.08–3.55)	**0.027**		
**t(4;14)**										
No	102	—				154	—			
Yes	5	4.76 (1.42–16.0)	**0.011**			15	1.41 (0.63–3.12)	0.4		
**t(14;16)**										
No	97	—				164	—			
Yes	10	1.05 (0.32–3.49)	>0.9			5	1.06 (0.26–4.40)	>0.9		
**Age**	107	0.98 (0.95–1.02)	0.4			169	0.99 (0.96–1.02)	0.5		
**High risk**										
No	84	—		—		118	—		—	
Yes	23	2.73 (1.28–5.83)	**0.009**	3.85 (1.60–9.26)	**0.003**	51	1.93 (1.11–3.37)	**0.02**	1.89 (1.03–3.45)	**0.043**
**EF < 45%**										
No	105	—				166	—			
Yes	2	2.54 (0.34–19.0)	0.4			3	1.99 (0.48–8.24)	0.3		
**CRS**										
No	53	—		—		31	—			
Yes	54	0.32 (0.14–0.73)	**0.007**	0.21 (0.08–0.55)	**<0.001**	138	0.82 (0.43–1.56)	0.5		
**Max CRS**	107	1.36 (0.32–5.71)	0.7			169	0.79 (0.42–1.48)	0.5		
**ICANS**										
No	91	—		—		135	—			
Yes	16	2.45 (1.04–5.79)	**0.041**	3.02 (1.09–8.35)	**0.043**	34	2.01 (1.08–3.74)	**0.027**		
**Max ICANS**	107	0.31 (0.11–0.89)	**0.029**			169	0.34 (0.17–0.69)	**0.003**	0.51 (0.24–1.10)	0.1
**At Least VGPR**	107	0.06 (0.02–0.20)	**<0.001**	0.06 (0.02–0.20)	**<0.001**	169	0.3 (0.17–0.53)	**<0.001**	0.29 (0.16–0.52)	**<0.001**

Abbreviations: CI = Confidence Interval, HR = Hazard Ratio.

**Table 4:** Baseline characteristics, clinical, and safety outcomes of BDT-exposed patients receiving CAR T-cell therapy versus bispecific antibodies.

Characteristic	CAR-T N = 22 ^1^	Teclistamab N = 58 ^1^	*p*-Value ^2^
**Age**	64 (61, 69)	68 (61, 71)	0.5
**Race**			0.5
African American	6 (27%)	8 (14%)	
Asian	0 (0%)	3 (5.2%)	
Hispanic	0 (0%)	1 (1.7%)	
White	16 (73%)	46 (79%)	
**Sex**			0.6
Female	11 (50%)	24 (41%)	
Male	11 (50%)	34 (59%)	
**ECOG**			0.3
0	0 (0%)	8 (14%)	
1	16 (80%)	40 (69%)	
2	3 (15%)	5 (8.6%)	
3	1 (5.0%)	5 (8.6%)	
Unknown	2	0	
**Heavy Isotype**			0.96
IGA	4 (18%)	11 (19%)	
IGD	0 (0%)	1 (1.7%)	
IGG	14 (64%)	32 (55%)	
Light Chain Disease	4 (18%)	14 (24%)	
**Light Chain**			>0.9
Kappa	12 (55%)	30 (52%)	
Lambda	10 (45%)	28 (48%)	
**R-ISS stage**			0.6
1	7 (39%)	13 (26%)	
2	7 (39%)	24 (48%)	
3	4 (22%)	13 (26%)	
Unknown	4	8	
**PCL**			>0.9
No	22 (100%)	56 (97%)	
Yes	0 (0%)	2 (3.4%)	
**Amyloid**			0.3
No	21 (95%)	58 (100%)	
Yes	1 (4.5%)	0 (0%)	
**EF < 45%**			0.4
No	13 (93%)	55 (98%)	
Yes	1 (7.1%)	1 (1.8%)	
Unknown	8	2	
**EMD**			0.5
No	9 (41%)	31 (53%)	
Yes	13 (59%)	27 (47%)	
**Prior SCT**			**0.015**
No	0 (0%)	13 (22%)	
Yes	22 (100%)	45 (78%)	
**LOT**	7.50 (6.00, 10.00)	8.00 (6.00, 10.00)	0.6
**Deletion 17p**			0.10
No	21 (95%)	45 (79%)	
Yes	1 (4.5%)	12 (21%)	
Unknown	0	1	
**t(4;14)**			0.7
No	20 (91%)	53 (93%)	
Yes	2 (9.1%)	4 (7.0%)	
Unknown	0	1	
**t(14;16)**			0.3
No	19 (86%)	54 (95%)	
Yes	3 (14%)	3 (5.3%)	
Unknown	0	1	
**Cytogenetic risk group**			0.8
High-risk	5 (23%)	16 (28%)	
Standard-risk	17 (77%)	42 (72%)	
**Bortezomib**			**0.038**
Exposed	7 (32%)	13 (23%)	
NOT Exposed	2 (9.1%)	0 (0%)	
Refractory	13 (59%)	44 (77%)	
Unknown	0	1	
**Carfilzomib**			0.8
Exposed	1 (4.5%)	3 (5.2%)	
NOT Exposed	2 (9.1%)	3 (5.2%)	
Refractory	19 (86%)	52 (90%)	
**Lenalidomide**			0.8
Exposed	1 (4.5%)	5 (8.8%)	
NOT Exposed	0 (0%)	2 (3.5%)	
Refractory	21 (95%)	50 (88%)	
Unknown	0	1	
**Pomalidomide**			>0.9
Exposed	1 (4.5%)	2 (3.4%)	
NOT Exposed	1 (4.5%)	3 (5.2%)	
Refractory	20 (91%)	53 (91%)	
**anti-CD38**			0.073
Exposed	2 (9.1%)	0 (0%)	
Refractory	20 (91%)	58 (100%)	
**Triple-class refractory**	17 (77%)	53 (91%)	0.13
**Penta-class refractory**	10 (45%)	34 (59%)	0.3
**Product**			**<0.001**
Cilta-cel	10 (45%)	0 (0%)	
Ide-cel	12 (55%)	0 (0%)	
Teclistamab	0 (0%)	58 (100%)	
**Previous BCMA-Directed Therapy**			**<0.001**
ADC	12 (54.5%)	12 (20.7%)	
Bispecific Antibody	9 (41%)	6 (10.3%)	
CAR-T	0 (0%)	29 (50%)	
Combination	1 (4.5%)	11 (19%)	
**CrCl < 45**			0.4
No	21 (95%)	51 (88%)	
Yes	1 (4.5%)	7 (12%)	
**CRS**			0.13
0	7 (32%)	28 (48%)	
1	12 (55%)	28 (48%)	
2	3 (14%)	2 (3.4%)	
**Hospitalization (days)**	11.0 (9.0, 16.0)	8.0 (7.0, 11.0)	**0.018**
Unknown	0	1	
**Acute Infection**			**0.005**
Unknown	0	2	
No	19 (86%)	28 (48%)	
Yes	3 (14%)	28 (48%)	
**Best Response**			0.38
sCR	0 (0%)	6 (11%)	
CR	4 (18%)	9 (16%)	
VGPR	7 (32%)	10 (18%)	
PR	3 (14%)	5 (8.8%)	
SD	3 (14%)	6 (11%)	
PD	5 (23%)	21 (37%)	
Unknown	0	1	
**Progression**	15 (68%)	35 (60%)	0.5
**Death**	8 (36%)	29 (50%)	0.3
**Median follow-up** (months)	16.4 (11.5, 21.8)	15.6 (12.4, 17.6)	—
**ANC Day 30**			**<0.001**
0	7 (32%)	33 (66%)	
1	1 (4.5%)	6 (12%)	
2	5 (23%)	4 (8.0%)	
3	4 (18%)	7 (14%)	
4	5 (23%)	0 (0%)	
Unknown	0	8	
**HgB Day 30**			**0.048**
0	3 (14%)	23 (46%)	
1	6 (29%)	12 (24%)	
2	8 (38%)	11 (22%)	
3	4 (19%)	4 (8.0%)	
Unknown	1	8	
**PLT Day 30**			**0.002**
0	4 (19%)	21 (42%)	
1	4 (19%)	16 (32%)	
2	2 (9.5%)	8 (16%)	
3	2 (9.5%)	2 (4.0%)	
4	9 (43%)	3 (6.0%)	
Unknown	1	8	
**ANC Day 90**			0.7
0	12 (63%)	26 (74%)	
1	3 (16%)	2 (5.7%)	
2	3 (16%)	5 (14%)	
3	0 (0%)	1 (2.9%)	
4	1 (5.3%)	1 (2.9%)	
Unknown	3	23	
**HgB Day 90**			**0.003**
0	4 (21%)	20 (57%)	
1	7 (37%)	10 (29%)	
2	8 (42%)	2 (5.7%)	
3	0 (0%)	3 (8.6%)	
Unknown	3	23	
**PLT Day 90**			**0.030**
0	7 (37%)	18 (51%)	
1	4 (21%)	13 (37%)	
2	3 (16%)	4 (11%)	
3	2 (11%)	0 (0%)	
4	3 (16%)	0 (0%)	
Unknown	3	23	
**ICANS**			>0.9
0	20 (91%)	52 (90%)	
1	1 (4.5%)	2 (3.4%)	
2	1 (4.5%)	2 (3.4%)	
3	0 (0%)	1 (1.7%)	
4	0 (0%)	1 (1.7%)	

^1^ Median (Q1, Q3); n (%). ^2^ Mann-Whitney U test; Fisher’s Exact Test for Count Data with simulated *p*-value (based on 2000 replicates); Fisher’s exact test. Continuous variables are reported as median (interquartile range) and were compared using the Mann-Whitney U test; categorical variables are reported as n (%) and were compared using Fisher’s exact test or the chi-square test as appropriate. *p*-values < 0.05 are considered statistically significant and shown in bold. High-risk cytogenetic abnormalities (HRCAs) were defined as del(17p), t(4;14), or t(14;16) detected at any time prior to therapy initiation. Triple-class refractory: refractory to a proteasome inhibitor, an immunomodulatory drug, and an anti-CD38 monoclonal antibody. Penta-class refractory: refractory to ≥2 proteasome inhibitors, ≥2 immunomodulatory drugs, and an anti-CD38 monoclonal antibody. BDT, BCMA-directed therapy; ECOG, Eastern Cooperative Oncology Group performance status; R-ISS, Revised International Staging System; CrCl, creatinine clearance; LOT, lines of therapy; SCT, stem cell transplant; EMD, extramedullary disease. Prior BCMA-directed exposure was categorized as prior CAR-T, antibody-drug conjugate (belantamab mafodotin), bispecific antibody, or combination.

**Table 5:** Univariate and multivariable Cox analysis of overall survival in the combined BDT-exposed cohort.

	Univariate			Multivariable		
Characteristic	N	HR (95% CI)	*p*-Value	N	HR (95% CI)	*p*-Value
**Type of treatment**			0.062			
Bispecific	57	—				
CAR-T	22	0.44 (0.18–1.07)				
**EMD**			0.057	79		0.3
No	39	—			—	
Yes	40	1.89 (0.97–3.68)			1.47 (0.75–2.90)	
**CrCl < 45**			0.3			
No	72	—				
Yes	7	1.73 (0.61–4.93)				
**R-ISS stage**			0.9			
1	20	—				
2	30	0.73 (0.33–1.62)				
3	17	0.85 (0.34–2.08)				
unknown	8	0.89 (0.28–2.78)				
**ECOG (0–1 vs. 2–3)**	77	0.5 (0.24–1.08)	0.1			
**Prior transplants**			0.075			
No	13	—				
Yes	66	0.48 (0.22–1.02)				
**LOT**	79	1.02 (0.92–1.14)	0.7			
**Deletion 17p**			0.7			
No	66	—				
Yes	13	0.84 (0.33–2.16)				
**t(4;14)**			0.2			
No	73	—				
Yes	6	0.33 (0.05–2.41)				
**t(14;16)**			0.2			
No	73	—				
Yes	6	2.05 (0.72–5.85)				
**Age**	79	1 (0.97–1.03)	>0.9			
**High risk**			0.8			
No	58	—				
Yes	21	1.1 (0.51–2.34)				
**EF < 45%**			0.15			
No	67	—				
Yes	4	2.77 (0.83–9.26)				
**CRS**			0.8			
No	35	—				
Yes	44	0.9 (0.47–1.73)				
**Max CRS**	79	1.49 (0.36–6.21)	0.6			
**ICANS**			0.14			
No	72	—				
Yes	7	2.1 (0.84–5.23)				
**Max ICANS**	79	0.35 (0.11–1.15)	0.13			
**At Least VGPR**	79	0.19 (0.08–0.41)	**<0.001**	79	0.2 (0.09–0.45)	**<0.001**

Abbreviations: CI = Confidence Interval, HR = Hazard Ratio.

**Table 6:** Treatment-stratified univariate and multivariable Cox analysis of overall survival in BDT-exposed patients.

	Bispecific, N = 57					CAR-T, N = 22		
	Univariate			Multivariable		Univariate		
Characteristic	N	HR (95% CI)	*p*-Value	HR (95% CI)	*p*-Value	N	HR (95% CI)	*p*-Value
**EMD**								
No	30	—		—		9	—	
Yes	27	3.35 (1.55–7.24)	**0.002**	3.21 (1.36–7.55)	**0.007**	13	0.54 (0.13–2.18)	0.4
**CrCl < 45**								
No	51	—				21	—	
Yes	6	1.71 (0.59–4.93)	0.3			1	0 (0.00–Inf)	>0.9
**R-ISS stage**								
1	13	—				7	—	
2	23	0.66 (0.27–1.62)	0.4			7	0.68 (0.11–4.07)	0.7
3	13	0.71 (0.25–1.99)	0.5			4	1.11 (0.18–7.08)	>0.9
unknown	8	0.68 (0.21–2.19)	0.5					
**ECOG (0–1 vs. 2–3)**	57	0.28 (0.13–0.62)	**0.002**	0.84 (0.31–2.30)	0.7	20	NE	>0.9
**LOT**	57	1.03 (0.92–1.15)	0.6			22	0.93 (0.70–1.23)	0.6
**Deletion 17p**								
No	45	—				21	—	
Yes	12	0.77 (0.29–2.01)	0.6			1	0 (0.00–Inf)	>0.9
**t(4;14)**								
No	53	—				20	—	
Yes	4	0.4 (0.05–2.96)	0.4			2	0 (0.00–Inf)	>0.9
**t(14;16)**								
No	54	—				19	—	
Yes	3	1.28 (0.30–5.41)	0.7			3	15.7 (1.42–175)	**0.025**
**Age**	57	1 (0.96–1.03)	0.8			22	0.99 (0.91–1.08)	0.8
**High risk**								
No	41	—				17	—	
Yes	16	0.82 (0.35–1.92)	0.6			5	3.48 (0.60–20.2)	0.2
**EF < 45%**								
No	54	—				13	—	
Yes	3	2.52 (0.59–10.8)	0.2			1	4.79 (0.43–53.8)	0.2
**CRS**								
No	28	—				7	—	
Yes	29	0.7 (0.34–1.45)	0.3			15	4.44 (0.54–36.5)	0.2
**Max CRS**	57	0.54 (0.13–2.29)	0.4			22	NE	>0.9
**ICANS**								
No	51	—				21	—	
Yes	6	2.58 (0.98–6.80)	0.056			1	0.77 (0.08–7.81)	0.8
**Max ICANS**	57	0.27 (0.08–0.89)	**0.031**	0.11 (0.02–0.58)	**0.016**	22	NE	>0.9
**At Least VGPR**	57	0.13 (0.05–0.34)	**<0.001**	0.11 (0.04–0.32)	**<0.001**	22	0.34 (0.07–1.79)	0.2

Abbreviations: CI = Confidence Interval, HR = Hazard Ratio, NE, not estimable owing to complete separation.

## Data Availability

Data is available upon reasonable request from the corresponding author.

## Supplementary Materials

The following supporting information can be downloaded at: https://www.mdpi.com/article/10.3390/cancers18193147/s1, Figure S1: Progression-free and overall survival with cilta-cel versus bispecific antibodies in the BDT-naïve cohort; Figure S2: Overall response rate according to prior BCMA-directed modality and washout interval in the BDT-exposed cohort; Figure S3: Progression-free and overall survival with cilta-cel versus bispecific antibodies in the BDT-exposed cohort; Figure S4: Inverse probability of treatment weighting- and doubly robust-adjusted progression-free and overall survival in the BDT-naïve cohort; Table S1: Univariate and multivariable Cox analysis of progression-free survival in the combined BDT-naïve cohort; Table S2: Treatment-stratified univariate and multivariable Cox analysis of progression-free survival in BDT-naïve patients; Table S3: Day-30 landmark analyses of progression-free and overall survival; Table S4: Restricted mean survival time analyses of overall survival; Table S5: Univariate and multivariable Cox analysis of progression-free survival in the combined BDT-exposed cohort; Table S6: Treatment-stratified univariate and multivariable Cox analysis of progression-free survival in BDT-exposed patients; Table S7: Covariate balance before and after inverse probability of treatment weighting.
