# Supplementary material for: Sequencing BCMA-Directed Therapies in Relapsed/Refractory Multiple Myeloma: A Real-World Comparison of CAR T-Cell Therapy and Bispecific Antibodies
Source: Cancers (Basel). 2026 Sep 29;18(19):3147. doi: 10.3390/cancers18193147 (PMC13664241; doi:10.3390/cancers18193147)
Supplement: Supplementary file 1 [file cancers-18-03147-s001.zip › cancers-4541167-supplementary.pdf]

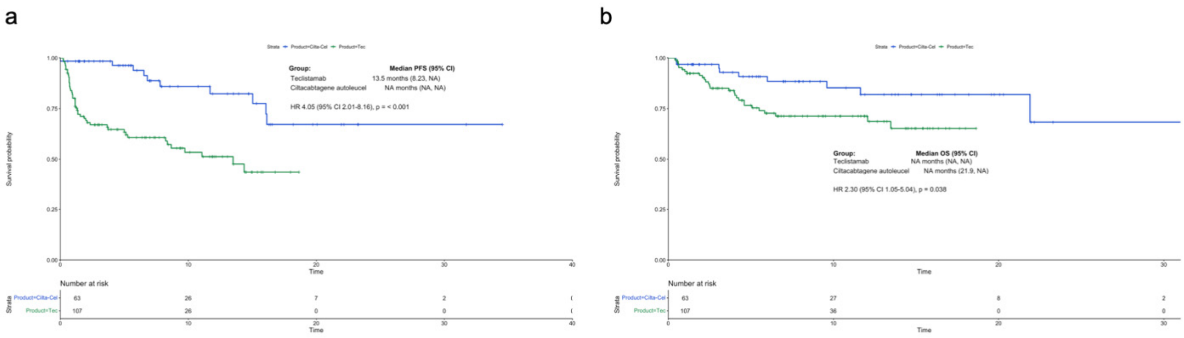

Figure S1. Subgroup analysis of ciltacabtagene autoleucel (cilta-cel) versus bispecific antibodies in BDT-naïve patients. Kaplan-Meier curves comparing cilta-cel (n=63) with BsAbs (n=107) among BDT-naïve patients. (a) Progression-free survival: PFS was not reached in the cilta-cel arm versus 13.5 months in the BsAb arm (HR 4.04 for BsAb vs cilta-cel, 95% CI 2.01–8.16; p < 0.001). (b) Overall survival: cilta-cel demonstrated superior OS compared with BsAbs (HR 2.30 for BsAb vs cilta-cel, 95% CI 1.05–5.04; p = 0.038). Median OS was not reached in either arm.

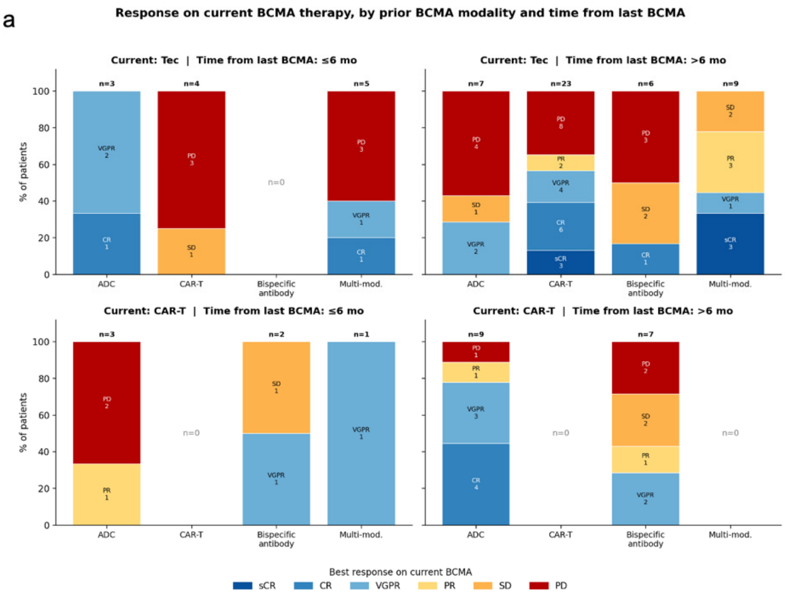

Figure S2. Overall response rate stratified by washout period and modality of prior BCMA-directed therapy. Heatmap displaying ORR in the BDT-exposed cohort according to the prior BCMA modality (CAR-T, antibody-drug conjugate [ADC, belantamab mafodotin], or BsAb) and the time interval (≤6 months vs >6 months) between the prior BDT and the index treatment.

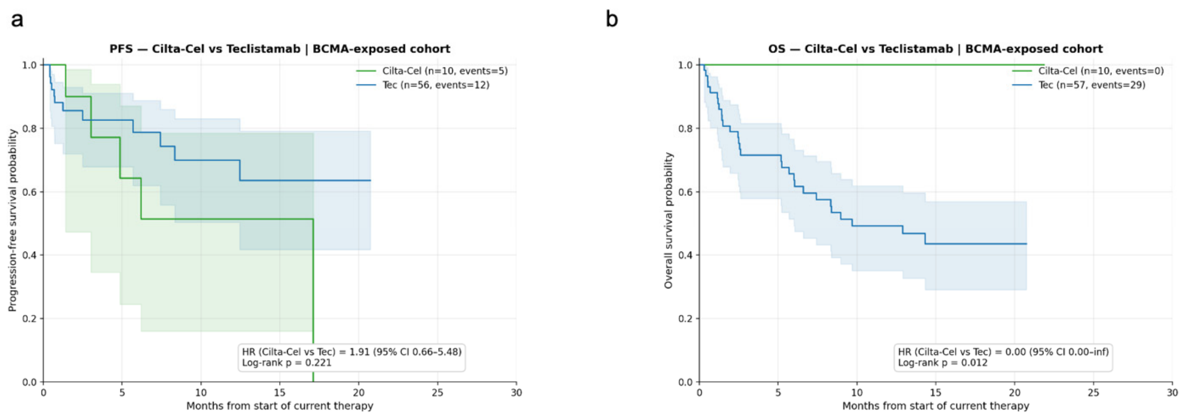

Figure S3. Subgroup analysis of cilta-cel versus bispecific antibodies in BDT-exposed patients. Kaplan-Meier curves comparing cilta-cel (n=10) with BsAbs (n=56 for PFS, n=57 for OS) among BDT-exposed patients. (a) Progression-free survival (HR 1.90 for BsAb vs cilta-cel, 95% CI 0.66–5.48; log-rank p = 0.22). (b) Overall survival; the Cox hazard ratio could not be estimated because no deaths occurred in the cilta-cel group. Tick marks indicate censored observations.

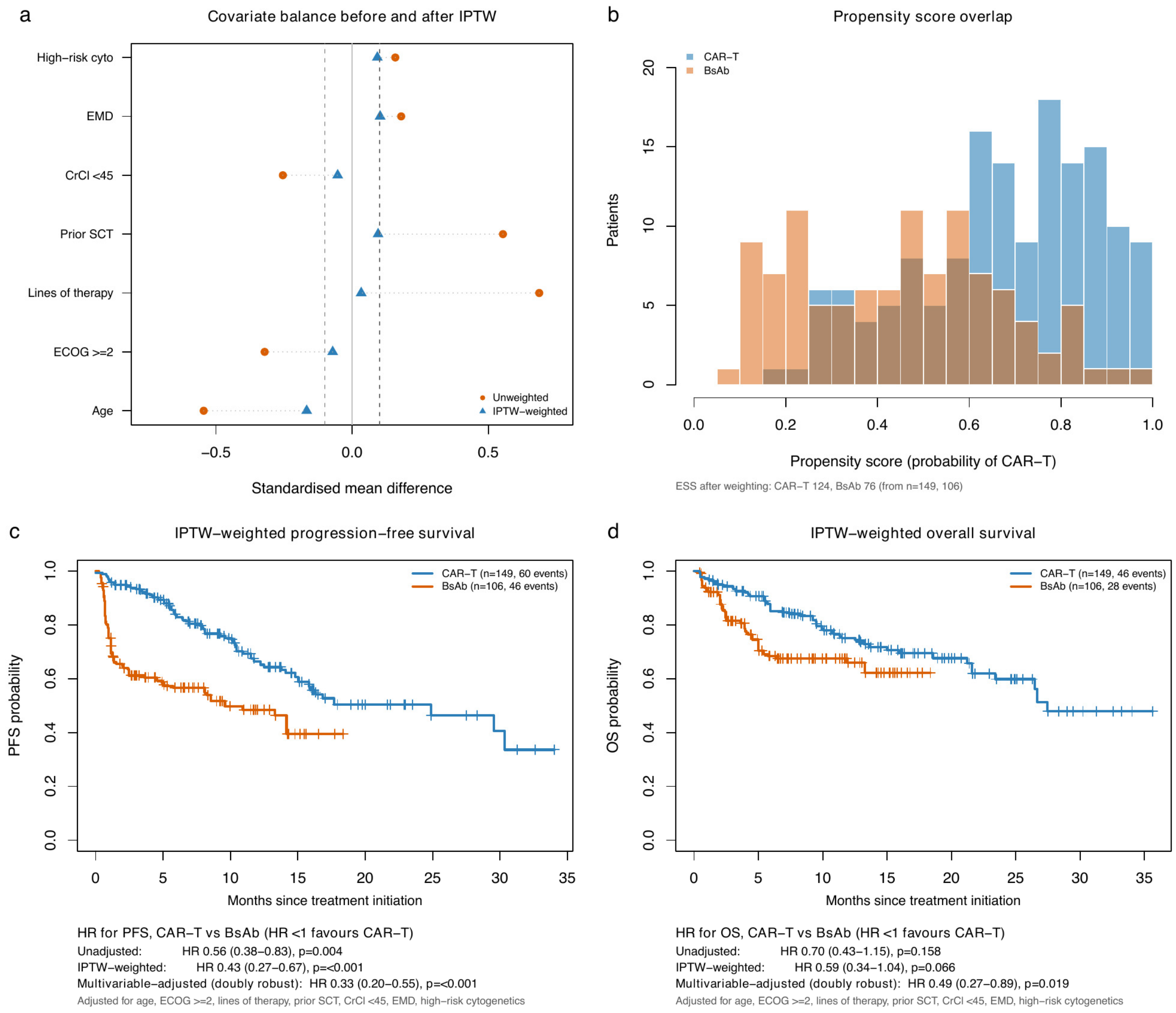

Figure S4. Inverse probability of treatment weighting (IPTW) analysis of CAR-T versus bispecific antibodies in BDT-naïve patients. (a) Standardized mean differences (SMD) before and after weighting; dashed lines denote the |SMD| < 0.10 balance threshold. Five of seven covariates were balanced after weighting, including the largest baseline imbalance, prior lines of therapy (SMD 0.687 to 0.033). **(b)** Propensity score distribution by treatment arm, demonstrating adequate overlap. **(c)** IPTW-weighted Kaplan-Meier curves for progression-free survival and **(d)** overall survival. Unadjusted, IPTW-weighted, and multivariable-adjusted (doubly robust) hazard ratios are shown beneath each curve; a hazard ratio <1 favours CAR-T. Tick marks indicate censored observations.

	Univariate				Multivariate			
**Characteristic**	**N**	**HR**	**95% CI**	**p-value**	**N**	**HR**	**95% CI**	**p-value**
**Type of treatment**				**0.007**	276			**<0.001**
Bispecific	107	—	—			—	—	
CAR-T	169	0.59	0.40, 0.86			0.41	0.26, 0.66	
**EMD**				**0.004**	276			0.11
No	202	—	—			—	—	
Yes	74	1.77	1.21, 2.59			1.39	0.93, 2.08	
**CrCl <45**				0.4				
No	239	—	—					
Yes	37	1.25	0.73, 2.12					
**R-ISS stage**				0.14	276			0.3
1	52	—	—			—	—	
2	105	0.9	0.53, 1.54			1.07	0.61, 1.87	
3	67	1.09	0.62, 1.92			0.99	0.54, 1.81	
unknown	52	0.56	0.29, 1.10			0.63	0.32, 1.24	
**ECOG (0-1 vs 2-3)**	259	0.92	0.52, 1.62	0.8				
**Prior transplants**				0.8				
No	79	—	—					

Yes	197	0.96	0.63, 1.47					
**LOT**	276	1.09	1.01, 1.17	**0.028**	276	1.23	1.14, 1.34	**<0.001**
**Deletion 17p**				0.2				
No	229	—	—					
Yes	47	1.38	0.88, 2.17					
**t(4;14)**				0.3				
No	256	—	—					
Yes	20	1.46	0.78, 2.71					
**t(14;16)**				0.5				
No	261	—	—					
Yes	15	1.3	0.63, 2.67					
**Age**	276	0.98	0.96, 1.00	0.11	276	0.97	0.95, 0.99	**0.011**
**High risk**				**0.017**	276			**<0.001**
No	202	—	—			—	—	
Yes	74	1.62	1.10, 2.38			2.29	1.50, 3.50	
**EF<45%**				0.5				
No	271	—	—					
Yes	5	1.6	0.51, 5.06					
**CRS**				**0.019**	276			**0.002**
No	84	—	—			—	—	
Yes	192	0.63	0.43, 0.92			0.5	0.33, 0.76	

**Max CRS**	276	1.36	0.81, 2.28	0.2				
**ICANS**				0.2				
No	226	—	—					
Yes	50	1.37	0.87, 2.18					
**Max ICANS**	276	0.67	0.37, 1.21	0.2				
**At Least VGPR**	276	0.2	0.14, 0.30	**<0.001**	276	0.12	0.08, 0.19	**<0.001**
Abbreviations: CI = Confidence Interval, HR = Hazard Ratio								

Table S1. Univariate and multivariable Cox proportional-hazards analysis of progression-free survival in the combined BDT-naïve cohort. Hazard ratios (HRs) with 95% confidence intervals (CIs) and corresponding p-values are shown for each covariate. The multivariate model was age-adjusted and included treatment type (CAR-T vs BsAb), HRCA status, EMD, CRS, ICANS, best response of ⩾VGPR, and lines of therapy. Statistically significant covariates ($p < 0.05$) are shown in bold. CAR-T was associated with improved PFS (HR 0.41, 95% CI 0.26–0.66, $p < 0.001$). Reference categories: treatment = BsAb; HRCA = standard risk; CRS/ICANS = absent; ⩾VGPR = no. CRS, cytokine release syndrome; ICANS, immune effector cell-associated neurotoxicity syndrome; LOT, lines of therapy.

	Bispecific, N = 107				CAR-T, N = 169						
	Univariate				Univariate				Multivariate		
**Characteristic**	**N**	**HR**	**95% CI**	**p-value**	**N**	**HR**	**95% CI**	**p-value**	**HR**	**95% CI**	**p-value**
**EMD**											0.065
No	84	—	—		118	—	—		—	—	
Yes	23	1.77	0.93, 3.36	0.082	51	2.01	1.25, 3.25	**0.004**	1.67	0.97, 2.86	
**CrCl <45**											
No	86	—	—		153	—	—				
Yes	21	1.75	0.88, 3.46	0.11	16	0.67	0.27, 1.67	0.4			
**R-ISS stage**											
1	23	—	—		29	—	—				
2	40	0.71	0.32, 1.59	0.4	65	1.12	0.53, 2.36	0.8			
3	29	1.01	0.45, 2.29	>0.9	38	1.22	0.56, 2.68	0.6			
unknown	15	0.86	0.33, 2.28	0.8	37	0.52	0.21, 1.32	0.2			
**ECOG (0-1 vs 2-3)**	107	0.89	0.44, 1.79	0.7	152	1.48	0.54, 4.09	0.4			
**Prior transplants**											
No	48	—	—		31	—	—				
Yes	59	1.27	0.70, 2.30	0.4	138	1.04	0.54, 1.99	>0.9			
**LOT**	107	1.11	0.97, 1.26	0.12	169	1.15	1.04, 1.27	**0.005**	1.2	1.07, 1.34	**0.002**

**Deletion 17p**											
No	96	—	—		133	—	—				
Yes	11	1.28	0.54, 3.03	0.6	36	1.69	0.99, 2.90	0.057			
**t(4;14)**											
No	102	—	—		154	—	—				
Yes	5	2.01	0.62, 6.50	0.2	15	1.48	0.71, 3.09	0.3			
**t(14;16)**											
No	97	—	—		164	—	—				
Yes	10	0.73	0.26, 2.07	0.6	5	2.37	0.86, 6.52	0.1			
**Age**	107	0.97	0.94, 1.00	0.1	169	0.97	0.95, 1.00	0.053			
**High_risk**											
No	84	—	—		118	—	—		—	—	
Yes	23	1.4	0.73, 2.72	0.3	51	1.98	1.23, 3.21	**0.005**	2.47	1.48, 4.13	**<0.001**
**EF<45%**											
No	105	—	—		166	—	—				
Yes	2	1.26	0.17, 9.18	0.8	3	1.64	0.40, 6.70	0.5			
**CRS**											
No	53	—	—		31	—	—				
Yes	54	0.59	0.33, 1.06	0.078	138	0.88	0.50, 1.55	0.7			
**Max CRS**	107	1.23	0.44, 3.42	0.7	169	1.26	0.69, 2.31	0.5			
**ICANS**											

No	91	—	—		135	—	—				
Yes	16	1.53	0.74, 3.18	0.3	34	1.36	0.75, 2.47	0.3			
**Max ICANS**	107	0.64	0.23, 1.79	0.4	169	0.6	0.29, 1.27	0.2			
**At Least VGPR**	107	0.06	0.03, 0.14	**<0.001**	169	0.39	0.23, 0.64	**<0.001**	0.2	0.11, 0.35	**<0.001**
Abbreviations: CI = Confidence Interval, HR = Hazard Ratio											

Table S2. Treatment-stratified univariate and multivariable Cox analysis of progression-free survival in BDT-naïve patients (CAR-T and BsAb arms analyzed separately). Cox proportional-hazards models were run separately for the CAR-T (n=169) and BsAb (n=107) arms. Hazard ratios, 95% CIs and p-values are reported; significant predictors ($p < 0.05$) are bold. In the CAR-T arm, increased LOT and HRCA predicted worse PFS while ⩾VGPR predicted improved PFS. In the BsAb arm, only ⩾VGPR was a significant predictor. Footer abbreviations as in Table 2.

	Overall survival					Progression-free survival				
**Cohort**	**N at risk**	**Events**	**HR (Tec vs Cilta-Cel)**	**95% CI**	**p-value**	**N at risk**	**Events**	**HR (Tec vs Cilta-Cel)**	**95% CI**	**p-value**
**Landmark Day 30**										
BCMA-naive	158	29	2.48	1.00, 6.12	0.049	145	34	2.86	1.31, 6.26	0.009
BCMA-exposed	62	24	NE	—	>0.9	50	27	1.08	0.41, 2.87	0.87

Table S3. Landmark analysis of progression-free and overall survival at Day 30 post CAR-T infusion, and 18-month survival estimates by treatment arm. To mitigate immortal-time bias inherent to comparisons involving CAR T-cell manufacturing, landmark analysis was performed at Day 30 post CAR-T infusion (or treatment initiation for BsAb). Patients who experienced an event or were censored before the landmark were excluded from the landmark analysis. Cox proportional-hazards models were fit on the landmark dataset; HRs are reported with 95% CIs and log-rank p-values. Cilta-cel demonstrated a higher PFS (HR 2.86, 95% CI 1.31–6.26, p = 0.009) and higher OS (HR 2.86, 95% CI 1.00–6.12, p = 0.049) versus BsAbs.

Parameter	BCMA-naive cohort (τ = 550 days)				BCMA-exposed cohort (τ = 630 days)			
	**Est.**	**SE**	**95% CI**	**p**	**Est.**	**SE**	**95% CI**	**p**
Restricted mean survival time (RMST), days								
BsAb	408.6	22.4	364.7 to 452.5	—	358.4	35.4	289.0 to 427.8	—
CAR-T	440.1	14.5	411.8 to 468.4	—	485.7	49.9	387.8 to 583.6	—
Restricted mean time lost (RMTL), days								
BsAb	141.4	22.4	97.5 to 185.3	—	271.6	35.4	202.2 to 341.0	—
CAR-T	109.9	14.5	81.6 to 138.2	—	144.3	49.9	46.4 to 242.2	—
Between-group contrast (BsAb vs CAR-T)								
RMST difference (BsAb − CAR-T), days	-31.5	—	−83.8 to 20.8	0.238	-127.3	—	−247.3 to −7.3	0.038
RMST ratio (BsAb / CAR-T)	0.928	—	0.819 to 1.052	0.245	0.738	—	0.558 to 0.976	0.033
RMTL ratio (BsAb / CAR-T)	1.287	—	0.859 to 1.926	0.221	1.882	—	0.912 to 3.886	0.087

τ, truncation time; RMST, restricted mean survival time; RMTL, restricted mean time lost; SE, standard error; CI, confidence interval; BsAb, bispecific antibody; CAR-T, chimeric antigen receptor T-cell therapy. RMST/RMTL and their difference are reported in days; ratios are dimensionless.

Table S4. Restricted mean survival time (RMST) analysis of overall survival in the BDT-naïve and BDT-exposed cohorts. RMST was used to summarize survival up to a pre-specified truncation time (τ) chosen as the latest follow-up point at which both arms had adequate at-risk numbers (τ = 550 days for the BDT-naïve cohort; τ = 630 days for the BDT-exposed cohort). RMST estimates are reported in days with standard errors (SE), 95% CIs, and the between-arm difference (BsAb − CAR-T) with corresponding 95% CI and p-value. A negative RMST difference favors CAR-T.

	Univariate				Multivariate			
**Characteristic**	**N**	**HR**	**95% CI**	**p-value**	**N**	**HR**	**95% CI**	**p-value**
**Type of treatment**				0.8				
Bi_specific	56	—	—					
CAR-T	22	0.93	0.51, 1.71					
**EMD**				0.11				
No	38	—	—					
Yes	40	1.58	0.90, 2.78					
**CrCl <45**				0.6				
No	71	—	—					
Yes	7	1.28	0.50, 3.27					
**R-ISS stage**				0.9				
1	20	—	—					

2	30	0.91	0.44, 1.90					
3	17	1.29	0.57, 2.92					
unknown	7	0.98	0.33, 2.93					
**ECOG (0-1 vs 2-3)**	76	0.57	0.29, 1.13	0.12				
**Prior transplants**				0.5				
No	12	—	—					
Yes	66	0.77	0.37, 1.59					
**LOT**	78	1.02	0.93, 1.13	0.6				
**Deletion 17p**				0.7				
No	65	—	—					
Yes	13	1.14	0.55, 2.38					
**t(4;14)**				0.8				
No	72	—	—					
Yes	6	1.11	0.44, 2.80					
**t(14;16)**				0.6				
No	72	—	—					
Yes	6	1.38	0.43, 4.48					
**Age**	78	0.97	0.94, 1.00	**0.03**	78	0.99	0.96, 1.02	0.6
**High risk**				0.6				
No	57	—	—					

Yes	21	1.18	0.63, 2.19					
**EF<45%**				0.5				
No	66	—	—					
Yes	4	1.56	0.48, 5.09					
**CRS**				0.7	78			>0.9
No	34	—	—			—	—	
Yes	44	0.9	0.51, 1.60			0.97	0.54, 1.72	
**Max CRS**	78	0.82	0.32, 2.07	0.7				
**ICANS**				0.2				
No	71	—	—					
Yes	7	1.85	0.78, 4.36					
**Max ICANS**	78	0.67	0.21, 2.18	0.5				
**At Least VGPR**	78	0.19	0.10, 0.36	**<0.001**	78	0.2	0.11, 0.39	**<0.001**
Abbreviations: CI = Confidence Interval, HR = Hazard Ratio								

Table S5. Univariate and multivariable Cox analysis of progression-free survival in the combined BDT-exposed cohort. Multivariate model adjusted for age and clinically relevant covariates. Best response of ≥VGPR was the only independent predictor of PFS (HR 0.20, 95% CI 0.11–0.39, $p < 0.001$).

	Bi_specific, N = 56							CAR-T, N = 22						
	Univariate				Multivariate			Univariate				Multivariate		
**Characteristic**	**N**	**HR**	**95% CI**	**p-value**	**HR**	**95% CI**	**p-value**	**N**	**HR**	**95% CI**	**p-value**	**HR**	**95% CI**	**p-value**
**EMD**														
No	29	—	—					9	—	—				
Yes	27	1.69	0.87, 3.30	0.12				13	1.42	0.47, 4.26	0.5			
**CrCl <45**														
No	50	—	—					21	—	—				
Yes	6	1.75	0.67, 4.57	0.3				1	0	0.00, Inf	>0.9			
**R-ISS stage**														
1	13	—	—					7	—	—				
2	23	0.97	0.41, 2.32	>0.9				7	0.69	0.15, 3.12	0.6			
3	13	1.48	0.55, 3.99	0.4				4	0.9	0.20, 4.08	0.9			
unknown	7	1.18	0.38, 3.69	0.8										
**ECOG (0-1 vs 2-3)**	56	0.41	0.18, 0.90	**0.027**	0.55	0.24, 1.24	0.2	20	1.02	0.27, 3.83	>0.9			
**LOT**	56	1.05	0.95, 1.17	0.3				22	0.89	0.71, 1.11	0.3			
**Deletion 17p**														
No	44	—	—					21	—	—				
Yes	12	1.27	0.57, 2.83	0.6				1	0.45	0.05, 3.79	0.5			
**t(4;14)**														

No	52	—	—					20	—	—				
Yes	4	1.64	0.57, 4.70	0.4				2	0.38	0.05, 3.11	0.4			
**t(14;16)**														
No	53	—	—					19	—	—				
Yes	3	1.01	0.14, 7.45	>0.9				3	1.88	0.41, 8.63	0.4			
**Age**	56	0.99	0.96, 1.02	0.4				22	0.89	0.83, 0.96	**0.002**	0.9	0.84, 0.97	**0.006**
**High risk**														
No	40	—	—					17	—	—				
Yes	16	1.28	0.62, 2.63	0.5				5	0.86	0.24, 3.12	0.8			
**EF<45%**														
No	53	—	—					13	—	—				
Yes	3	2.93	0.89, 9.67	0.078				1	0	0.00, Inf	>0.9			
**CRS**														
No	27	—	—					7	—	—		—	—	
Yes	29	1.21	0.62, 2.38	0.6				15	0.29	0.09, 0.88	**0.03**	0.42	0.12, 1.47	0.2
**Max CRS**	56	0.5	0.12, 2.11	0.3				22	1.12	0.31, 4.10	0.9			
**ICANS**														
No	50	—	—					21	—	—				
Yes	6	2.25	0.87, 5.84	0.095				1	0.68	0.08, 5.49	0.7			
**Max ICANS**	56	0.39	0.12, 1.28	0.12				22	74,962,252	0.00, Inf	>0.9			

**At Least VGPR**	56	0.13	0.06, 0.31	**<0.001**	0.14	0.06, 0.33	**<0.001**	22	0.42	0.15, 1.18	0.1				
Abbreviations: CI = Confidence Interval, HR = Hazard Ratio															

Table S6. Treatment-stratified univariate and multivariable Cox analysis of progression-free survival in BDT-exposed patients. Cox models stratified by treatment arm. In the BsAb arm, ⩾VGPR was the only significant predictor (HR 0.14, p < 0.001). In the CAR-T arm, age was the only significant predictor in the multivariate model (HR 0.90, 95% CI 0.84–0.97, p = 0.006). Footer abbreviations as in Table 2.

covariate	SMD unweighted	SMD weighted
Age	-0.545	-0.167
ECOG2	-0.321	-0.071
LOT	0.687	0.033
PriorSCT	0.554	0.095
CrCl45	-0.254	-0.053
EMD	0.18	0.103
HRCA	0.159	0.093

Table S7 shows the SMD prior to and after the Inverse probability treatment weighting. Covariates with SMDs between -0.1 and 0.1 were considered balanced.
